# Braided peridotite sills and metasomatism in the Rum Layered Suite, Scotland

**DOI:** 10.1007/s00410-019-1652-9

**Published:** 2020-01-30

**Authors:** Luke N. Hepworth, Felix E. D. Kaufmann, Lutz Hecht, Ralf Gertisser, Brian O’Driscoll

**Affiliations:** 1 School of Geography, Geology and the Environment, Keele University, Keele, Staffordshire, ST5 5BG UK; 2 Leibnitz-Institut für Evolutions- Und Biodiversitätsforschung, Museum für Naturkunde Berlin, Invalidenstrasse 43, 10115 Berlin, Germany; 3 Department of Earth and Environmental Sciences, University of Manchester, Oxford Road, Manchester, M13 9PL UK

**Keywords:** Harrisite, Layered intrusion, Sill emplacement, Infiltration metasomatism, Reactive liquid flow

## Abstract

The Rum Eastern Layered Intrusion (ELI; Scotland) is an open-system layered intrusion constructed of 16 macro-rhythmic units. Each of the macro-rhythmic units consists of a peridotite base and a troctolite (± gabbro) top, previously attributed to the fractional crystallisation of a single magma batch. This classic paradigm has been challenged, however, with evidence presented for the emplacement of peridotite sills in Units 9, 10, and 14, such as cross-cutting relationships, upward-oriented apophyses, and lateral discontinuities. To test whether the other major peridotites within the ELI represent sills, we have carried out new field, petrographic, and mineral chemical analyses of the peridotites in Units 7, 8 and 9. The peridotites display large- and small-scale cross-cutting relationships with the overlying troctolite, indicative of an intrusive relationship. The peridotites also show large-scale coalescence and lateral spatial discontinuities such that the ELI unit divisions become arbitrary. Harrisite layers and Cr-spinel seams found throughout Units 7, 8, and 9 suggest the peridotites were constructed incrementally via repeated injections of picritic magma. Our observations allow for distinct subtypes of peridotite to be defined, separated by intrusive contacts, allowing for their relative chronology to be determined. Older, poikilitic peridotite, rich in clinopyroxene, is truncated by younger, well-layered peridotite, containing abundant harrisite layers. In addition to the new peridotite subtypes defined in this study, we find strong evidence for laterally oriented metasomatism within clinopyroxene-rich wehrlites at the top of the Unit 8 peridotite. The wehrlites and surrounding peridotites record a complex series of metasomatic reactions that transformed thin picrite sills into clinopyroxene-rich wehrlites without any evidence for the sort of vertical melt movement typically posited in layered intrusions. The observations presented in this study from the ELI cannot be reconciled with the classic magma chamber paradigm and are better explained by the emplacement of composite sills into pre-existing feldspathic cumulate (gabbro or troctolite). The evidence for sill emplacement presented here suggests that the layered complex was constructed by a combination of sill emplacement and metasomatism, forming many of the unusual (often clinopyroxene-rich) lithologies that surround the sills. The broad-scale formation of the layered peridotites via incremental sill emplacement, suggested by the occurrence of upward-oriented apophyses, coalescence, and lateral discontinuity, could be applied to much larger ultramafic intrusions, which might have formed by similar mechanisms.

**Electronic supplementary material:**

The online version of this article (10.1007/s00410-019-1652-9) contains supplementary material, which is available to authorized users.

## Introduction

Layered intrusions offer an excellent opportunity to investigate the processes of magmatic differentiation and solidification within the upper crust, particularly regarding the formation of crystal mushes (Tepley and Davidson 2003; Holness 2005; Humphreys 2009; Tegner et al. 2009; O’Driscoll et al. 2007, 2009a; Humphreys and Holness 2010; Holness et al. 2013, 2015, 2017a; Leuthold et al. 2014; Namur et al. 2015; Hepworth et al. 2017, 2018). However, the mechanism by which layered intrusions form remains controversial and hotly debated (Latypov et al. 2015; Marsh 2015; Latypov 2019; Scoates et al. 2019). The traditional view of layered intrusions is as large bodies of crystal-poor magma that accumulate crystal mushes on the floor and margins of the chamber, formed principally by gravity-driven accumulation such as crystal settling, magmatic density currents, and slumping (e.g. Emeleus et al. 1996; McBirney and Nicolas 1997; Tegner et al. 2009; Maier et al. 2013; Holness et al. 2017a, b). This classic view has been challenged by some, particularly given the recent advancements in high-resolution geochronology, suggesting that many parts of layered intrusions formed semi-randomly by the emplacement of numerous sills (Bédard et al. 1988; Mungall et al. 2016; Wall et al. 2018). Understanding the fundamental construction mechanism of layered intrusions is particularly important when elucidating the magmatic processes operating during solidification. The calculated cooling histories for layered intrusions viewed as ‘magma chambers’ could have been severely disrupted by repeated emplacement of sills, oversimplifying a complex and protracted development (e.g. Cawthorn and Walraven 1998). Indeed, many of the lithologies comprising layered intrusions could record sill emplacement events, whereby repeated injection of fresh magma into a crystal mush has caused severe modification of primary features and created new, hybrid lithologies previously assumed to be related by magmatic fractionation (e.g. Leuthold et al. 2014).

In this study, we focus on some of the macro-rhythmic units that make up the Rum Eastern Layered Intrusion (ELI) in NW Scotland. The comparatively small size and young age of the ELI provides an ideal opportunity to examine both large- and small-scale features of a layered intrusion without some of the textural and chemical equilibration in larger layered intrusions (Tanner et al. 2014). The ELI is composed of up to 16 macro-rhythmic units, each consisting of a peridotite base and an overlying troctolite ± gabbro (Brown 1956; Volker and Upton 1990). The repeated nature of macro-rhythmic (or cyclic) units within layered intrusions is typically thought to represent individual pulses of magma that have fractionated into an upwardly evolving sequence of cumulate (e.g. Brown 1956; Tepley and Davidson 2003; Holness 2005; O’Driscoll et al. 2007; Brandiss et al 2014; Hunt et al. 2017; Latypov et al. 2017). The macro-rhythmic units in the ELI have traditionally been linked to the settling of successive phases of olivine, plagioclase, and clinopyroxene upon replenishment into the magma chamber (Brown 1956; Emeleus et al. 1996). However, this model was first challenged by Bédard et al. (1988) upon the detailed examination of some ELI peridotites (chiefly Units 9 and 10). The authors showed conclusively that both peridotite bodies represented sills emplaced into pre-existing gabbroic cumulate. It was speculated, based upon their investigation, that many (if not all) of the peridotites represented sills, echoing Harker’s (1908) original interpretation. A recent investigation of Unit 10 provided further support for this argument, with emphasis on the role of small volume (incremental) replenishment (Hepworth et al. 2017). Although the role of sill emplacement has been recognised in at least two of the macro-rhythmic units of the ELI, its wider applicability has yet to be established, with important implications for the formation of the layered intrusion.

We present new field, petrographic, and mineral chemical data from the peridotites of Units 7, 8, and 9 of the ELI. Our observations reveal both large- and small-scale cross-cutting relationships between the peridotites and overlying allivalite (a local term for plagioclase cumulate), indicative of sill emplacement. Many of the peridotites also coalesce, pointing to a braided or anastomosing geometry, further suggesting sill emplacement. All the peridotites studied here contain at least two subtypes, providing important insights into the relative chronology of emplacement. Our data also highlight the key role of incremental construction of the peridotites where harrisite layers, displaying features such as upward-oriented apophyses, point to the repeated injection of small-volume picrite sills in well-layered peridotites. The presence of numerous Cr-spinel seams found throughout these peridotites provide further support for this process. An important finding of our study is that the macro-rhythmic units of Units 7–10 are merely an arbitrary juxtaposition of peridotite sills and pre-existing feldspathic cumulate (e.g. gabbro or troctolite). Furthermore, we have recognised two additional peridotite subtypes in the Unit 8 peridotite which have undergone extensive, laterally oriented, metasomatism, transforming primary lithologies into new, clinopyroxene-rich varieties. By recognising these additional subtypes, we can place tighter constraints on the relative chronology of complex sequences of crystal mushes, many layers of which may represent metasomatic reactions between a pre-existing lithology and younger sills (e.g. Hepworth et al. 2018).

## Geological background

**Fig. 1:**
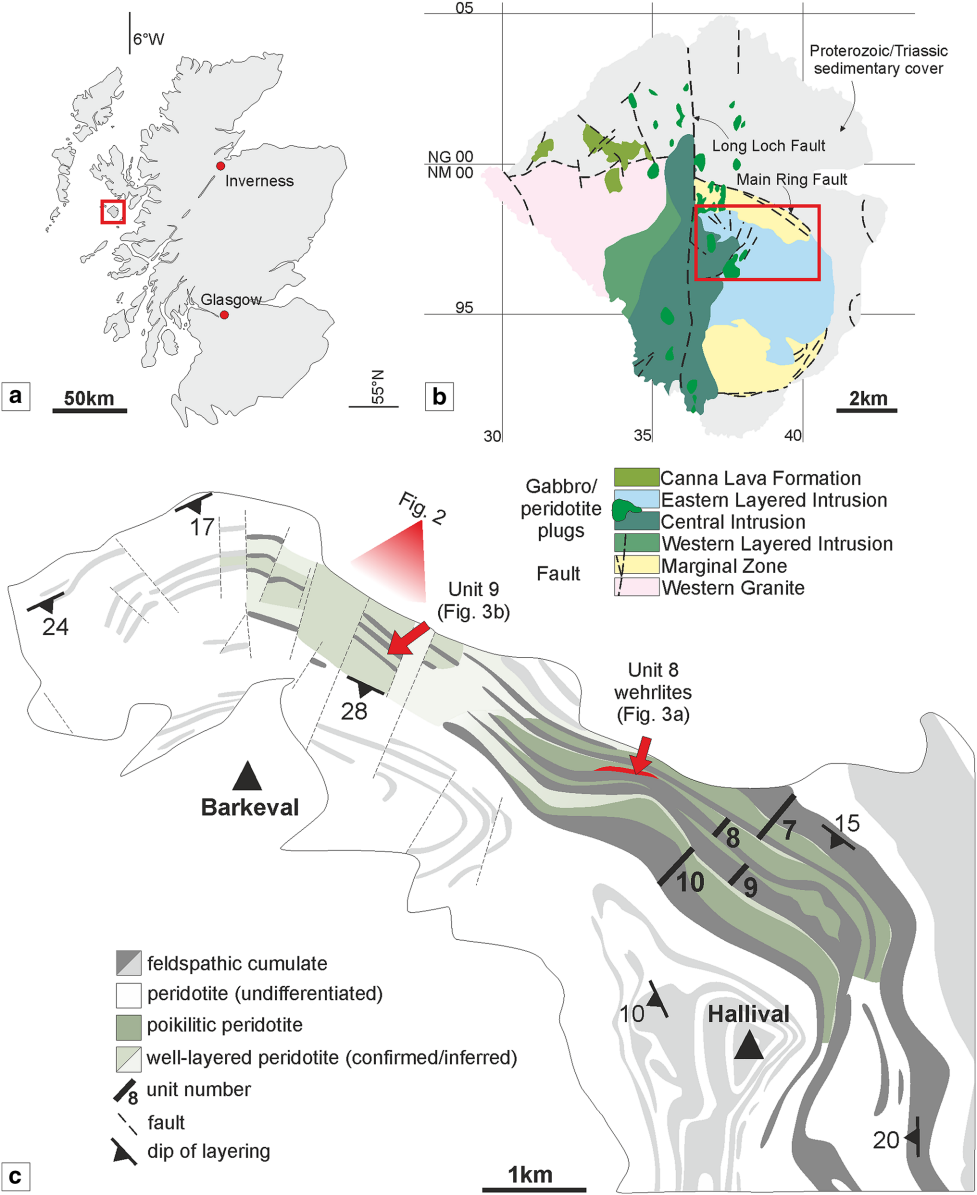
**a**. Map of Scotland and location of the Isle of Rum (red box). **b** Simplified geological map of the Isle of Rum. **c** Geological map of the northern limb of the ELI with simplified geological map of the peridotites and allivalites in this study. After Emeleus 1994

The Rum Igneous Complex was formed as a part of the British and Irish Palaeogene Igneous Province, an area of extensive magmatism along the north-western coast of the British Isles, during the opening of the North Atlantic at ~ 60 Ma (Hamilton et al. 1998; Emeleus et al. 1996; Fig. 1a). The Rum Layered Suite (RLS) can be divided into three distinct portions: the Western Layered Intrusion (WLI), the Eastern Layered Intrusion (ELI), and the Central Intrusion (CI) (Fig. 1b). The WLI is composed of a thick sequence of layered peridotites which generally dip shallowly towards the east (Wadsworth 1961; Hepworth et al. 2018), including the type locality for the skeletal-olivine textured peridotite ‘harrisite’ (Harker 1908; Wager et al. 1960; Wadsworth 1961; Donaldson 1974). The ELI consists of 16 macro-rhythmic units, each consisting of a peridotite base, and a troctolite ± olivine gabbro top that dips shallowly towards the west (Brown 1956; Volker and Upton 1990; Fig. 1b). The CI is a diverse portion of the RLS, consisting of a wide variety of plagioclase and olivine cumulates, displaying a wide variety of dip orientations, including convolute-style layering (Volker and Upton 1990). The entire complex is bisected by the north–south trending Long Loch Fault (LLF), the presumed feeder conduit for the layered intrusion (Emeleus et al. 1996). The parental magmas of the RLS are generally considered to be picritic, carrying a variable cargo of olivine phenocrysts (Greenwood et al. 1990; Upton et al. 2002; Leuthold et al. 2015). The ELI has long been regarded as a type example of open-system magmatism, being constructed of multiple pulses of magma, as opposed to a single, closed-system pulse (e.g. Skaergaard Intrusion, East Greenland; Wager and Deer 1939). Radiogenic isotope studies from several of the macro-rhythmic units (e.g. Rb–Sr, Re–Os) lend support to this hypothesis (e.g. Palacz 1984, 1985; O’Driscoll et al. 2009b), with more focused petrological studies emphasising the open-system behaviour within individual units (Palacz and Tait 1985; Renner and Palacz 1987; Tepley and Davidson 2003; Holness and Winpenny 2008). Of these studies, the nature of emplacement (and growth of the intrusion) has putatively been via aggradation (i.e. forming bottom to top) within a large body of crystal-poor magma (e.g. Dunham and Wadsworth 1978; Faithfull 1985; Tait 1985; Emeleus et al. 1996; Holness 2005, 2007; Emeleus and Troll 2014). This paradigm was first challenged by Bédard et al. (1988) who established, based on field evidence, that the Unit 9 and 10 peridotites represented sill-like intrusions, later corroborated for Unit 9 by textural and geochemical evidence in the overlying allivalite (Holness 2005; Holness et al. 2007; Leuthold et al. 2014). A recent investigation of the Unit 10 peridotite by Hepworth et al. (2017) supported an intrusive origin for this body, and showed that much of the peridotite was formed incrementally, via the intrusion of numerous thin sills (represented by harrisite layers). A similar interpretation was applied to the WLI (Hepworth et al. 2018). Despite the lack of layer continuity typical of sills in the ELI and WLI, intrusive peridotite is likely a major component of the CI (Volker and Upton 1990), contrary to the classic model (e.g. Emeleus and Troll 2014).

## Field relationships

**Fig. 2:**
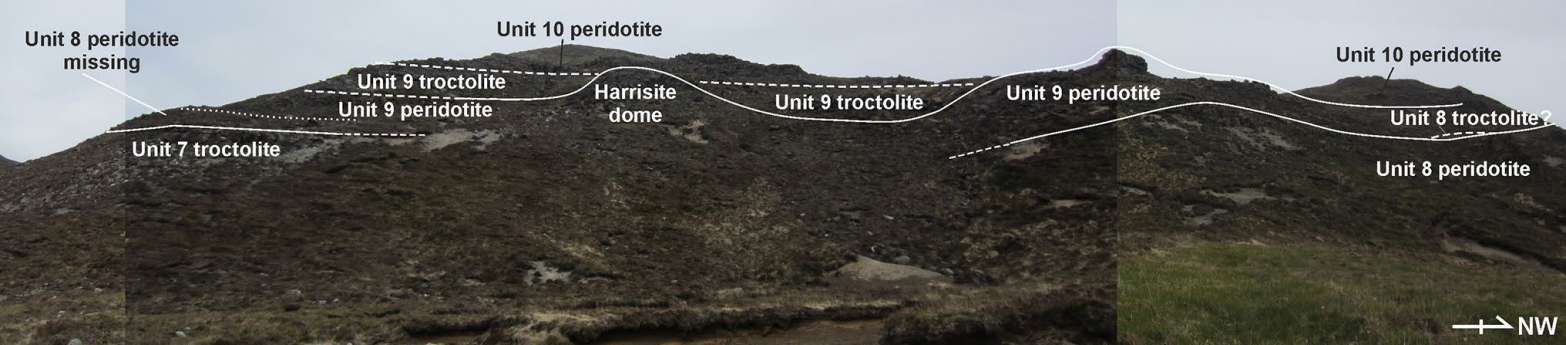
Field relationships between Units 7, 8, and 9 around Barkeval, with well-layered peridotite coalescence and cross-cutting relationships. Note the dome-like upwelling of harrisite from the Unit 9 well-layered peridotite. Note also the absence of Unit 8 between the marker horizons of Units 7 and 9 to the left of the figure

Our study focused upon the exposures of peridotite within Units 7, 8, and 9, found between Barkeval and Hallival (Fig. 1c). We have chosen Units 7, 8, and 9 for their respective marker horizons mappable across the study area, e.g. the Unit 7 allivalite contains an unusually high volume of anorthosite schlieren and numerous well-oriented folds, while the Unit 9 allivalite contains the clinopyroxene ‘wavy horizon’ studied by Bédard et al (1988) and Holness et al (2007). The peridotite and allivalite pairs comprising the macro-rhythmic units first defined by Brown (1956) around Hallival are significantly less well ordered with distance northward (Fig. 1b, c). Many of the units appear to thin towards the north by tens of metres and are absent from the succession in some places (Fig. 2). The increase in peridotite volume relative to allivalite discussed by Bédard and Sparks (1991) has also been observed here. The peridotites can be subdivided into the ‘poikilitic peridotite’ and ‘well-layered peridotite,’ respectively, similar to the subtypes found in Unit 10 (Palacz and Tait 1985; Hepworth et al 2017). These subtypes are not ubiquitous across the study area, with one subtype typically dominating the north and south, respectively, e.g. the poikilitic peridotite is thickest in all three units in the south of the study area (i.e. around Hallival) but is notably absent in the north (i.e. around Barkeval) (Fig. 1c). The two subtypes share an invariable stratigraphic relationship where present together, with the poikilitic peridotite found overlying the well-layered peridotite. The subsequent field descriptions have been divided into the aforementioned subtypes, with additional (and previously unidentified) subtypes described within the top part of the Unit 8 peridotite, described under ‘other’.

### Poikilitic peridotites

**Fig. 3:**
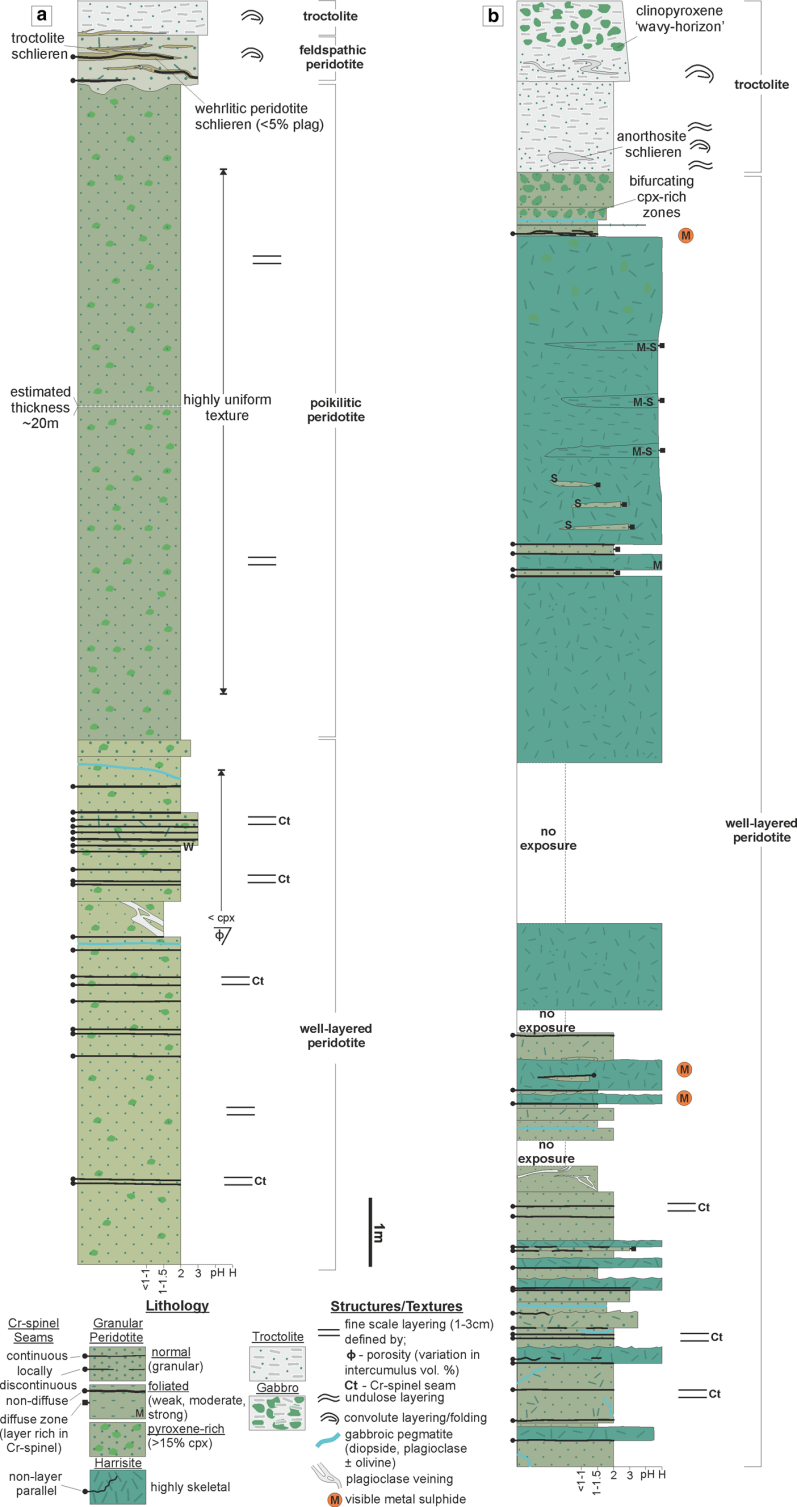
Graphic logs of the Units 8 and 9 peridotites. **a** Compiled sequence of the Unit 8 peridotite from across the study area. The log of the well-layered (Cr-spinel-bearing) peridotite was taken from around Barkeval, as the vertical exposure of well-layered peridotites is poor in the south (around Hallival). The poikilitic and upper section is from around Hallival (Fig. 1c). **b** The upper section of the Unit 9 well-layered peridotite around Barkeval (Fig. 1c). Note the wavy horizon marker found in the allivalite and clinopyroxene-rich zone at the very top of the peridotite

**Fig. 4:**
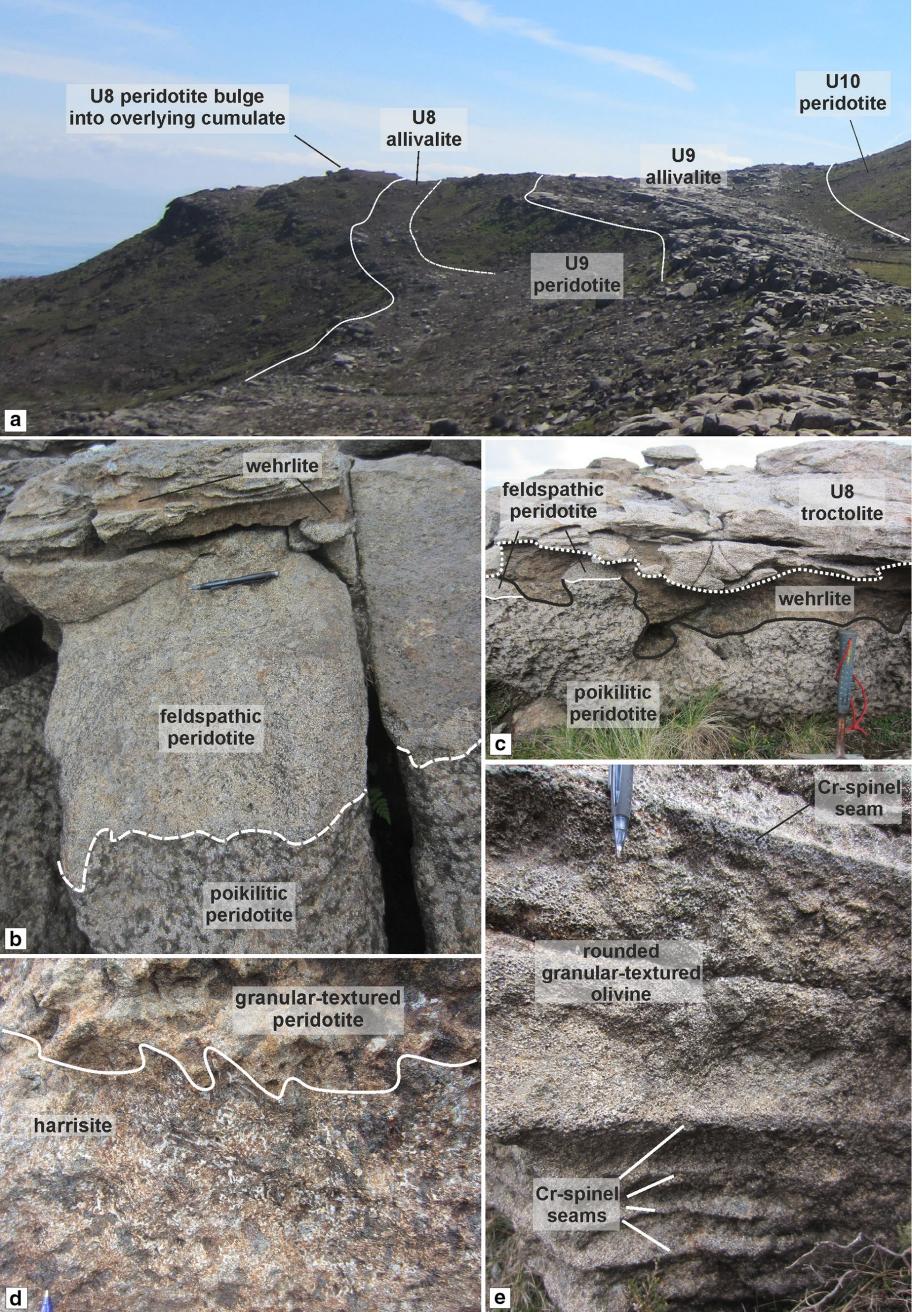
Field relationships of Unit 8. **a** Cross section of Units 8 and 9 with the Unit 8 peridotite bulge occurring at the same level as the overlying troctolite, potentially protruding into it. **b** Upper portion of the Unit 8 peridotite with three distinct peridotite types. Note the convolute nature of layering adjacent to the wehrlite. **c** Irregular boundary of the peridotites and overlying troctolite, with only small pods of the feldspathic peridotite and wehrlite delineating the troctolite and poikilitic peridotite. **d** Harrisite with irregular upper boundary with granular textured peridotite from the well-layered peridotites. Note the unusual abundance of blue-green clinopyroxene oikocrysts not observed in Unit 7 or 9 Cr-spinel seam in the wehrlite, with abundant green clinopyroxene oikocrysts. **e** Centimetre-sized Cr-spinel seams in the well-layered peridotite (see also Fig. 3a)

**Fig. 5:**
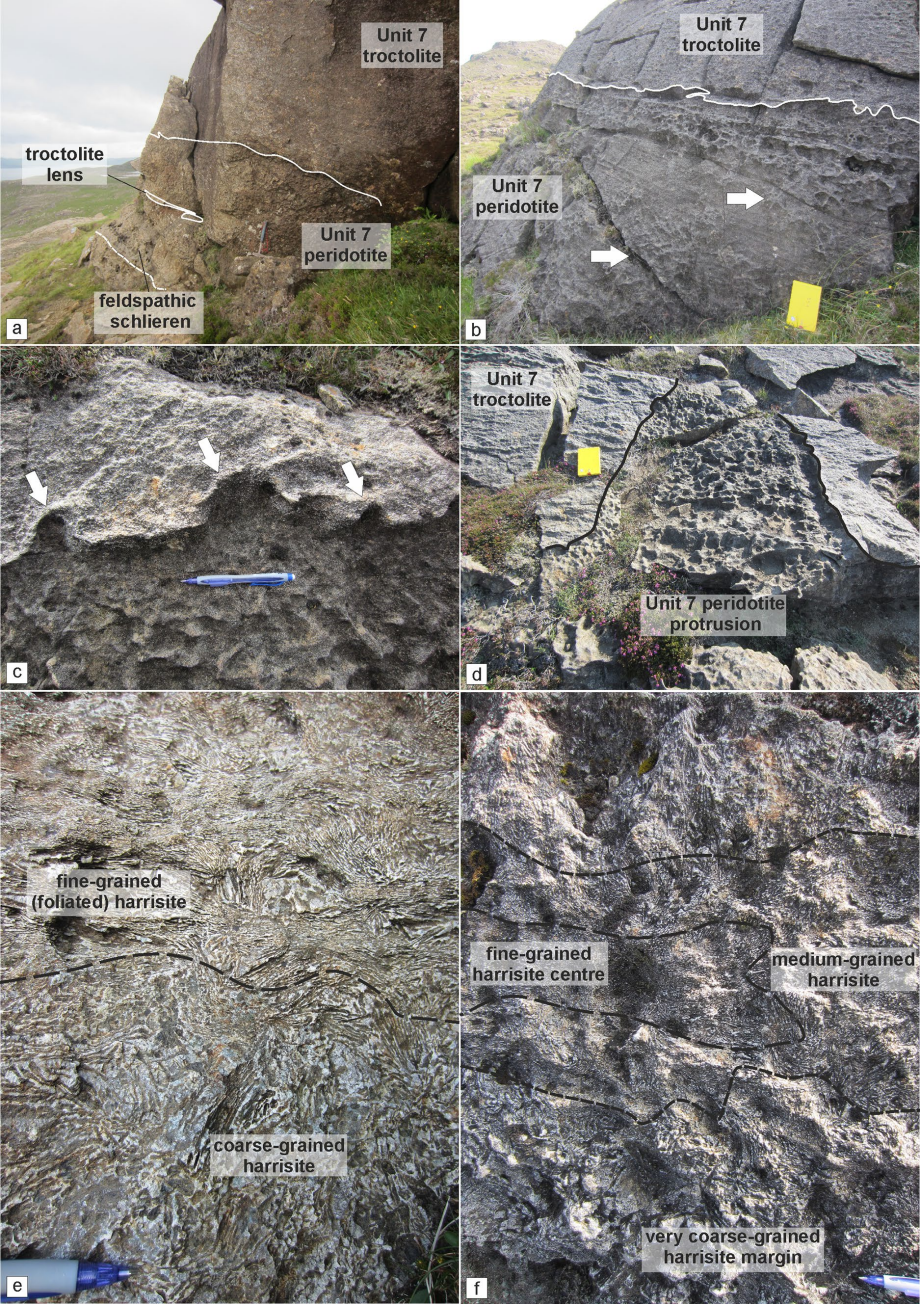
Field relationships of the Unit 7 peridotite. **a** Poikilitic peridotite at Hallival with a troctolite lens close to the boundary with the overlying troctolite. **b** Well-layered peridotite around Barkeval with undulose boundary with the overlying troctolite and patchy intercumulus variation (arrowed). Layering is defined by Cr-spinel seams **c** Small-scale peridotite apophyses into the troctolite. **d** Large-scale peridotite protrusion crossing the overlying troctolite. **e** Harrisite layer with foliated crystals in the upper section of the photograph. **f** Composite harrisite layer with fining inward sequence of harrisitic olivine

The poikilitic peridotite is only present in the south of the study area (i.e. around Hallival; Fig. 1c). It is typically tens of metres thick, massive, and highly uniform with only locally discontinuous schlieren of plagioclase defining some vague, sporadic layering (e.g. Fig. 3a). The poikilitic peridotite appears to thin towards the north before exposure becomes absent, similar to the observation made in Unit 10 (Hepworth et al. 2017). The boundaries with the overlying troctolite are sharp and broadly undulose, with large-scale cross-cutting relationships observed in Units 8 and 9 (Fig. 4a; Bédard et al. 1988). The boundary with the underlying well-layered peridotite is sharp. It is easily identified by the large, centimetre-size clinopyroxene oikocrysts (Fig. 4b, c). Autoliths of troctolite can be found in all three units, with smaller, elongate examples found within Unit 7 (Fig. 5a). More feldspathic patches of peridotite are noted where this autolith occurs within Unit 7 (Fig. 5a). The large, metre-size block of troctolite described by Bédard et al (1988) in their Fig. 11 from Unit 9 was observed in this study.

### Well-layered peridotites

**Fig. 6:**
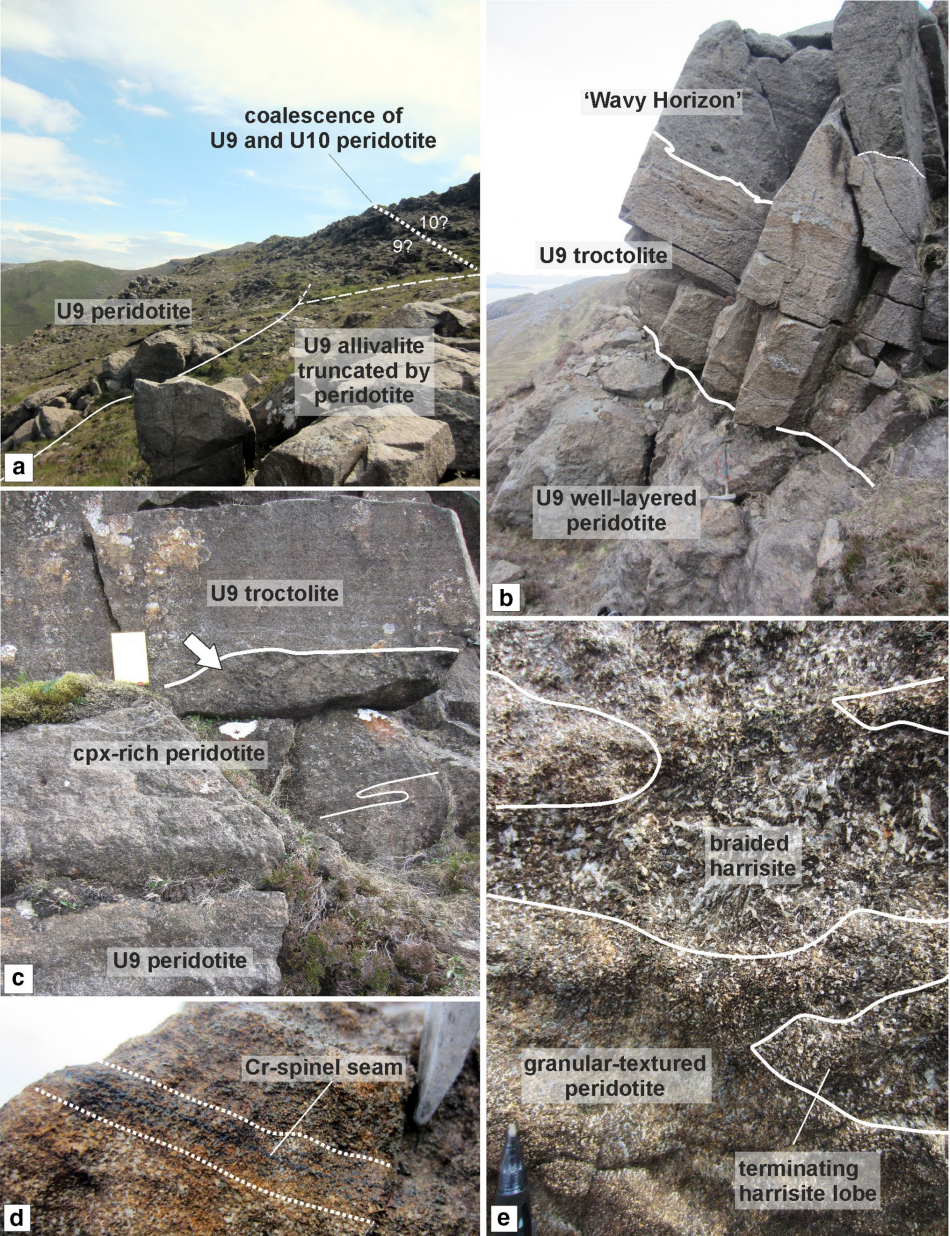
Field relationships of the Unit 9 peridotite. **a** Example of peridotite coalescence between the Unit 9 and 10 peridotites with complete truncation of the troctolite. **b** Well-layered peridotite in contact with the troctolite at Barkeval (including the wavy horizon marker). **c** Bifurcating clinopyroxene-rich zone at the top of the well-layered peridotite at Barkeval. Note the absence of clinopyroxene oikocrysts in the troctolite directly above this zone, suggesting impermeability. **d** Cr-spinel seam in the well-layered peridotite. **e** Braided/anastomosing harrisite layer and small terminating lobe of harrisite within the granular-textured peridotite

The well-layered peridotite is well exposed in the north of the study area (i.e. around Barkeval), with thinner packages of the well-layered peridotite in the south of the study area (i.e. around Hallival; Fig. 1c). The well-layered peridotites are characterised by the pronounced layering defined by granular-textured peridotite, harrisite, and Cr-spinel seams (Figs. 4d, e, 5b, e and 6d, e). The boundary with the overlying poikilitic peridotite and underlying allivalite is sharp (see Unit 10; Hepworth et al 2017). A boundary-type chromitite seam is present along some of the boundary between Units 7 and 8 (O’Driscoll et al 2010). In the north of the study area, where the poikilitic peridotite is absent, both small- and large-scale protuberances occur along the boundary with the overlying allivalite. Small-scale (5–10 cm) peridotite apophyses protrude the Unit 7 troctolite along the boundary (Fig. 3c), with much larger metre-scale protuberances along the strike (Fig. 3d). Large-scale protuberances (in order of several metres) also occur within the well-layered peridotite of Unit 9, penetrating into the overlying allivalite and the base of Unit 10 (Fig. 2). Coalescence is observed between Units 8 and 9, and 9 and 10, respectively. The allivalite is absent between Units 8 and 9 in numerous places between Hallival and Barkeval, with the two peridotites representing one indistinguishable body of rock (Fig. 2). The Unit 9 peridotite also truncates the overlying troctolite and connects (via a bridge) with the successive peridotite of Unit 10 (Fig. 6a). The well-layered peridotites are composed of intercalated harrisite and granular-textured peridotite, defining the type of layering alongside numerous Cr-spinel seams (Figs. 4d, e, 5b, e and 6d, e). The harrisite layers vary in thickness, but are typically < 1 m thick, with unusually thick layers observed towards the top of the Unit 9 peridotite (Fig. 3b). Harrisite layers from all three units studied here display upward-oriented apophyses, bifurcation, and lateral termination (Figs. 3b, 4d and 6e), similar to those layers described in Unit 10 (Hepworth et al 2017). Rafts and lenses of granular-textured peridotite are common within the thick harrisite layers (Fig. 3b). The harrisite layers are made up of coarse-grained skeletal and amoeboidal crystals, often in texturally heterogeneous layers. Many of the layers contain patches of varying olivine grain sizes and morphologies to that comprising the bulk of the layer. Foliation of elongated olivine crystals parallel to layering is observed in many harrisites (e.g. Fig. 3b). Texturally composite harrisite layers were observed in Units 7 and 9 (Fig. 5e, f). The skeletal olivine from harrisites in Unit 7 is particularly coarse-grained, with centimetre-sized crystals common, but with many layers displaying distinct zones of grain size, often fining towards the centre (Fig. 5f). These layers also display an alignment of elongated harrisite, with distinct undulations of aligned olivine crystals (Fig. 5e, f). The texture of granular-textured peridotite is very similar between each unit, consisting of fine-grained rounded olivine crystals, with the exception of parts of the Unit 8 peridotite found in the north of the study area, which contain unusually coarse-grained olivine (3–5 mm; Fig. 4e). The layering found within the granular-textured peridotite (i.e. not associated with harrisite) is defined by the presence of Cr-spinels (and some feldspathic or gabbroic veins) (Fig. 4e, 5b and 6d). An unusually clinopyroxene-rich peridotite layer occurs at the contact with the peridotite and troctolite within Unit 9, and appears to bifurcate along strike (Fig. 3b, 6c). It is laterally extensive across tens of metres and besides the increase in modal clinopyroxene is texturally identical to the underlying peridotite (Fig. 6c).

### Other peridotites

In addition to the two broad peridotite subtypes, two other subtypes, a feldspathic peridotite and a wehrlite, can be found at the very top of the Unit 8 peridotite around Hallival (Fig. 1c). A schematic representation of the complete sequence of the Unit 8 peridotite is illustrated in Fig. 3a.

The feldspathic peridotite is a distinctly clinopyroxene-poor peridotite that demarcates the peridotite from the overlying troctolite (Fig. 4a–c). The boundary with the overlying troctolite is sharp and broadly undulose—flat, whilst the boundary with the underlying poikilitic peridotite is sharp and undulose (Fig. 4b). The feldspathic peridotite is discontinuous along the strike, suggesting a complex topology (Fig. 4c). In the field, the appearance of the feldspathic peridotite differs only from the poikilitic peridotite by the reduced clinopyroxene abundance, with some convolute layering where it interacts with the wehrlite, described below (Fig. 4b, c).

**Fig. 7:**
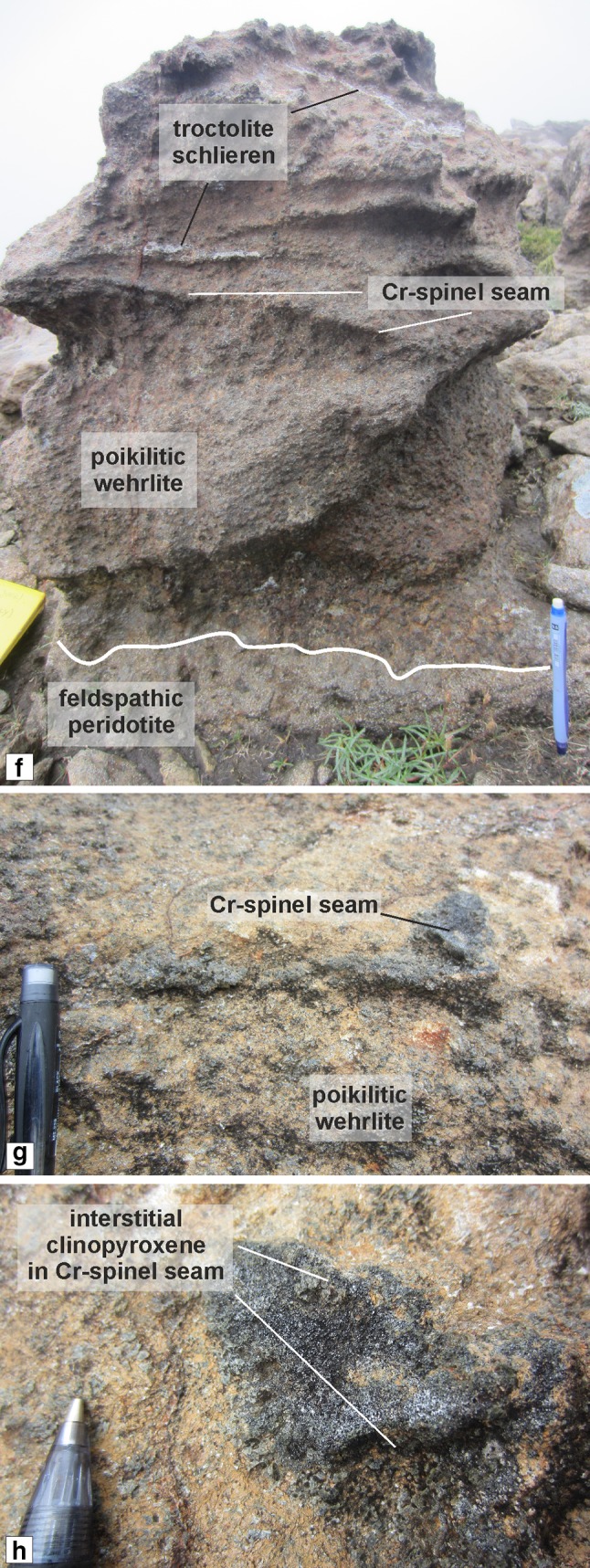
Field features of the Unit 8 wehrlites. **a** Thick wehrlite with prominent clinopyroxene oikocrysts, troctolite schlieren, and Cr-spinel seam. Note the reduced clinopyroxene contact of the underlying feldspathic peridotite. **b** Discontinuous clinopyroxene-hosted Cr-spinel seams in the wehrlite. **c** Magnified view of a clinopyroxene-rich Cr-spinel seam in the wehrlite

The wehrlite is present at the top of the peridotite package as sill-like bodies, lenses, and schlieren, varying in thickness from 10 to 50 cm (Fig. 4b, c). The bodies can be observed cross-cutting the feldspathic peridotite and overlying troctolite in numerous places (Fig. 5b, c). The wehrlite is distinctively dark coloured, reflecting the high clinopyroxene and olivine content, respectively, with the clinopyroxene occurring as larger, centimetre-sized oikocrysts with little to no plagioclase. Very thin (< 1 cm) feldspathic schlieren occur in the thicker wehrlite bodies (Fig. 7a). Cr-spinel stringers are also present in the wehrlite (Fig. 7b). The stringers are 5–10 mm thick and highly discontinuous, strictly confined to the wehrlite, and do not occur along the contact with the feldspathic peridotite. The stringers do not generally demarcate lithological/mineralogical variation. Clinopyroxene dominates the intercumulus proportion even within Cr-spinel stringers, with little to no plagioclase present (Fig. 7c).

## Petrography

### Poikilitic peridotites

**Fig. 8:**
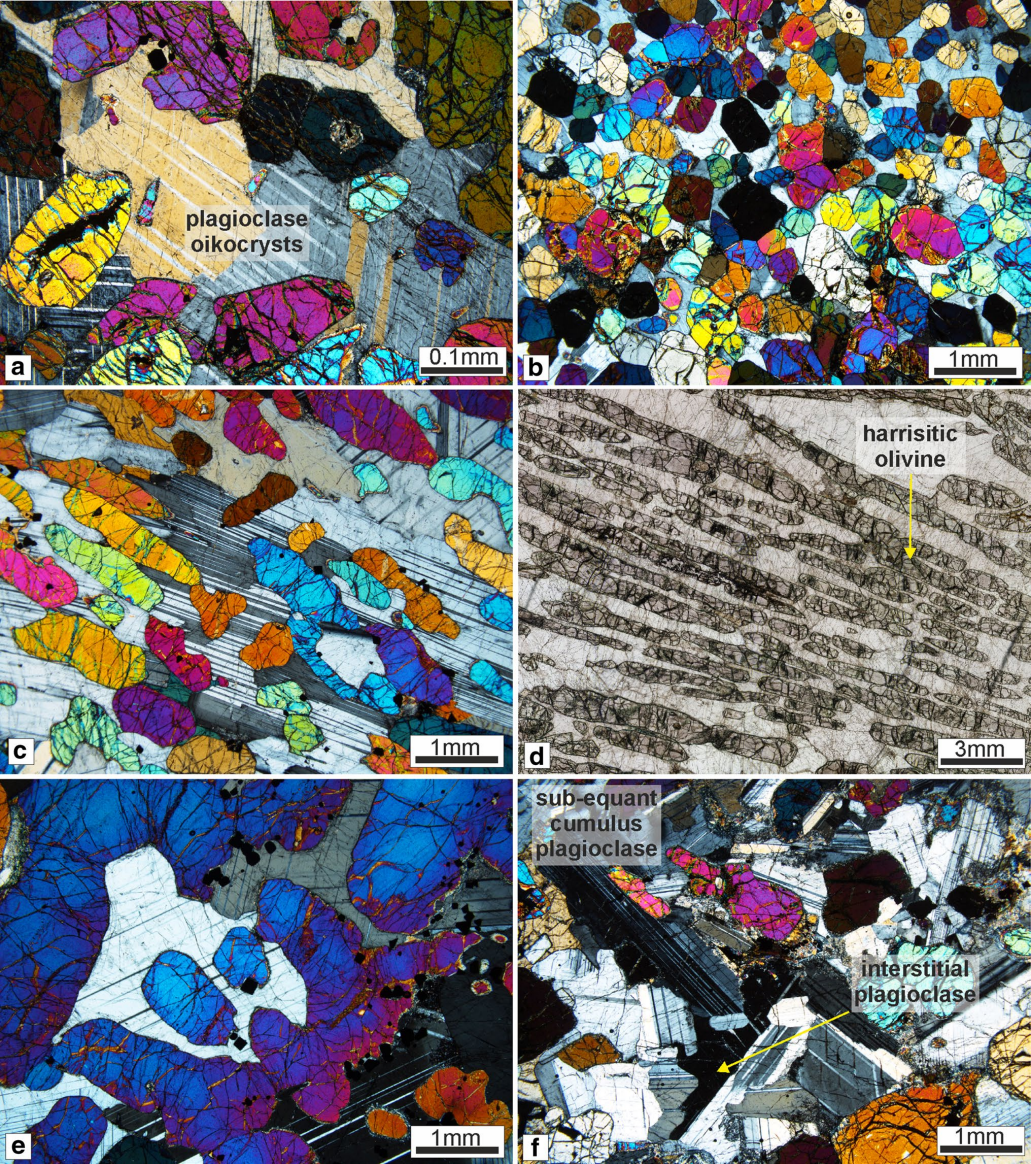
Petrography of the Units 7, 8, and 9 peridotites. **a** Typical intercumulus of plagioclase texture of in the Unit 8 poikilitic peridotite. **b** Fine-grained equant olivine from a layer in the Unit 9 well-layered peridotite. **c** Foliated skeletal and elongated olivine crystals from the Unit 9 well-layered peridotite. Compositional zoning of intercumulus plagioclase is also present. **d** Highly skeletal olivine in harrisite from Unit 7 well-layered peridotite. Many of the crystals in view are in optical continuity. **e** Large skeletal olivine in harrisite. The photomicrograph contains only a single olivine crystal. **f** Cumulus texture of plagioclase in the feldspathic peridotite of Unit 8 with some intercumulus crystals noted

The cumulus mineralogy of the poikilitic peridotites consists of olivine, comprising 60–70 vol% of the bulk rock. The olivine crystals are 1–3 mm, equant–elongate, with rarer skeletal and amoeboidal olivine (> 5 mm), often found in small patches. Non-penetrative foliation of olivine parallel to layering is observed across samples. Plagioclase dominates the intercumulus fraction (~ 60 vol%), consisting of coarse-grained subhedral oikocrysts (3–5 mm) with coarse-grained, rounded clinopyroxene oikocrysts (> 10 mm) (Fig. 8a). Patchy and oscillatory zoning is present in plagioclase oikocrysts, with weak patchy zoning also observed in some clinopyroxene oikocrysts. Cr-spinel occurs between the olivine framework, enclosed by plagioclase in accessory amounts (~ 3 vol%).

### Well-layered peridotites

Granular-textured peridotite in the well-layered peridotite is dominated by cumulus olivine, accounting for ~ 80 vol% of the bulk rock. The olivine is equant–rounded (1–3 mm) with uncommon skeletal and amoeboidal crystals (5–10 mm). Some of the granular-textured peridotites in Unit 8 are unusually coarse-grained and rounded (3–5 mm), particularly with proximity to Cr-spinel seams. Layers of granular-textured peridotite within Unit 9 are particularly fine grained (< 1 mm; Fig. 8b). Elongate and amoeboidal olivine is found more commonly with proximity to Cr-spinel seams in Unit 9, where a weak–moderate foliation is also observed (Fig. 8c). Very fine-grained (< 0.3 mm) euhedral Cr-spinel crystals occur within olivine embayments. Rounded inclusions of brown amphibole and plagioclase are observed in larger olivine crystals. The intercumulus mineralogy is dominated by plagioclase with < 10% clinopyroxene. Both phases are subhedral, sub-rounded, and > 3 mm in size. The mineralogy of harrisites is the same as that of granular-textured peridotites, with cumulus olivine and intercumulus plagioclase (± clinopyroxene) in sub-equant proportions. Intercumulus clinopyroxene, where present, is anhedral, but can comprise up to 10 mm-sized crystals, as well as rinds around coarser-grained olivine crystals. Patchy zoning (and oscillatory zoning) is present within plagioclase (Fig. 8b). Olivine morphology is dominated by coarse- to very coarse-grained, skeletal and amoeboidal shapes (> 10 mm; Fig. 8d, e), with similarly coarse-grained, sub-equant plagioclase oikocrysts (Fig. 8e). The abundance of clinopyroxene oikocrysts is elevated in both granular-textured peridotites and harrisites in Unit 8 (e.g. Fig. 4d). Secondary amphibole, biotite, chlorite, and serpentine are found as trace phases in all peridotites, but noticeably less abundant in Unit 9. Cr-spinel seams in the well-layered peridotite exhibit a chain-textured configuration with olivine similar to those described by O’Driscoll et al. (2010).

### Other peridotites

The cumulus mineralogy of the Unit 8 feldspathic peridotite consists of both olivine and plagioclase (Fig. 8f). Olivine is coarse- to very coarse-grained (2–5 mm) and dominated by anhedral, amoeboidal morphologies with subordinate equant crystals (Fig. 8f), while cumulus plagioclase is typically < 1 mm, tabular, and euhedral. Zoning is observed in cumulus plagioclase, including oscillatory zoning. Intercumulus clinopyroxene is uncommon, and where found occurs as subhedral and coarse-grained oikocrysts (5–10 mm) (akin to those in the poikilitic peridotite). Intercumulus plagioclase occurs rarely between cumulus plagioclase in anhedral crystal shapes. Cr-spinel can be found in trace amounts (< 2 vol%), typically within olivine embayments. Large, centimetre-size secondary biotite crystals were observed alongside trace amounts of chlorite, calcite, and epidote.

**Fig. 9:**
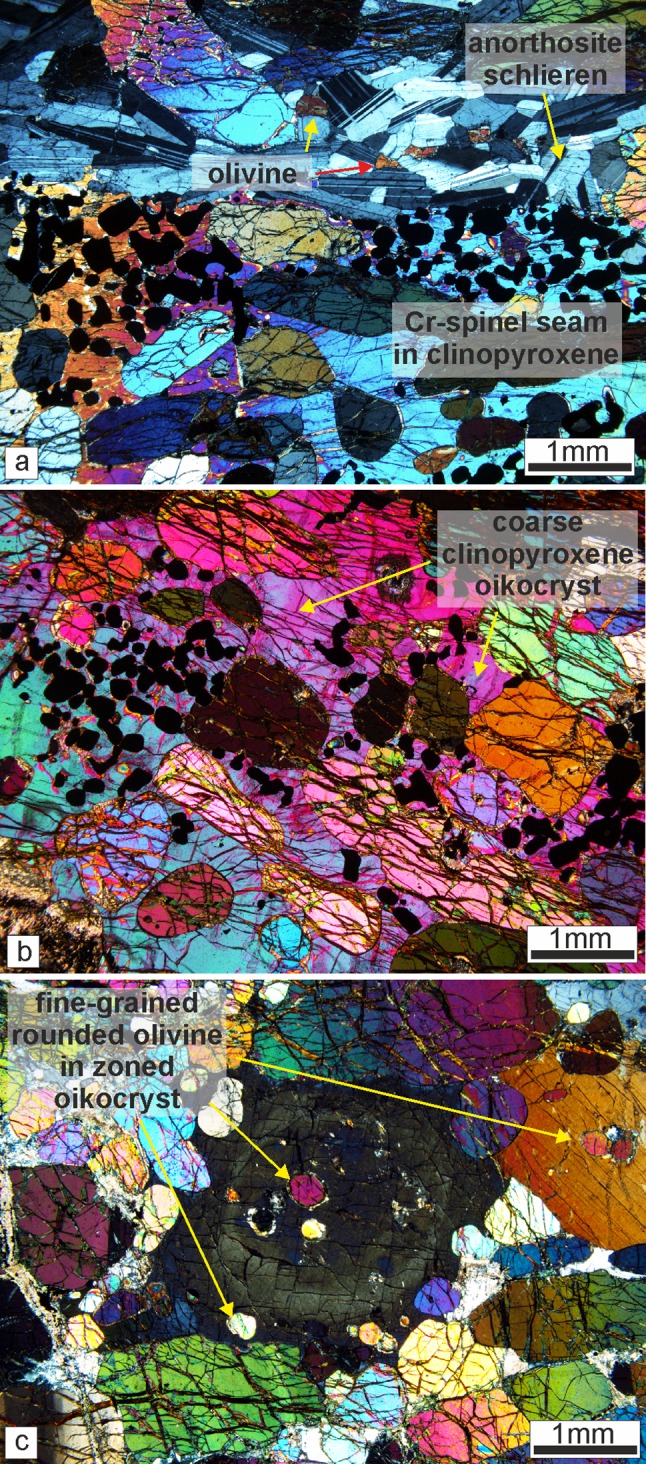
Petrography of the Unit 8 wehrlites. **a** Cr-spinel seam with anorthosite schlieren containing cumulus textured plagioclase in wehrlite. Note the size of the clinopyroxene oikocryst in the bottom of the image. **b** Clinopyroxene-hosted Cr-spinel seam in wehrlite. Note the single, large oikocryst across the whole photomicrograph. **c** Zoned clinopyroxene oikocryst in wehrlite with small rounded olivine crystals

The cumulus mineralogy of the wehrlite is exclusively olivine, dominated by equant crystals with subordinate elongated shapes (~ 0.5–2 mm). Individual olivine crystals can display a texture whereby the crystals embay surrounding crystals (i.e. cumulus) and also fill interstitial space (i.e. intercumulus). This is most apparent close to Cr-spinel seams and anorthosite schlieren, where olivine is also noticeably finer grained (Fig. 9a). Clinopyroxene occupies > 90 vol% of the intercumulus space as large, centimetre-sized oikocrysts (Fig. 9b, c). Patchy and oscillatory zoning are common in clinopyroxene (Fig. 9c). Olivine is also significantly reduced in size where it is enclosed by large clinopyroxene oikocrysts. The small included crystals are often in optical continuity (Fig. 9c). Plagioclase is very rare, but becomes marginally more abundant near Cr-spinel seams, where it is fine grained and anhedral (< 0.5 mm). Plagioclase crystals in an anorthosite schlieren found above a Cr-spinel seam displayed in Fig. 7e are notably fine grained and tabular (cumulus in texture) and weakly aligned. Cr-spinel seams are ubiquitously hosted within clinopyroxene with subhedral–anhedral Cr-spinel crystals (i.e. amoeboidal) (Fig. 9a, b). Composite textures are present in some seams found in the wehrlite, where the core of the seam is coarser grained than the margins (see Fig. 11b). Base metal sulphides are abundant throughout the wehrlite. Trace abundances of secondary minerals such as amphibole (brown/green), biotite, epidote, and calcite are present, with above trace abundances of chlorite and serpentine.

## Mineral chemistry

### Methodology

Mineral chemical data in this study were obtained using a JEOL JXA-8900RL EMP at the Geowissenschafliches Zentrum der Universität Göttingen (GZG) in 2014, and a JEOL JXA-8500F EMP at the Museum für Naturkunde Berlin in 2017. The details of the analysis conditions can be found in Electronic Supplementary Material 1. Ferric iron content of Cr-spinel was calculated assuming perfect stoichiometry following Droop (1987). Small variations in ferric iron should therefore be treated with caution (Quintiliani et al. 2006). To compare the coalescence of well-layered peridotites, our study focuses on the well-layered peridotites in Units 7, 8, and 9, found in the north of the study area (i.e. around Barkeval; Fig. 1c). Given the complexity of the Unit 8 peridotite documented in this study, we present the mineral chemical characteristics of each peridotite subtype (e.g. poikilitic, well-layered, feldspathic, and wehrlite).

### Results

#### Olivine

**Fig. 10:**
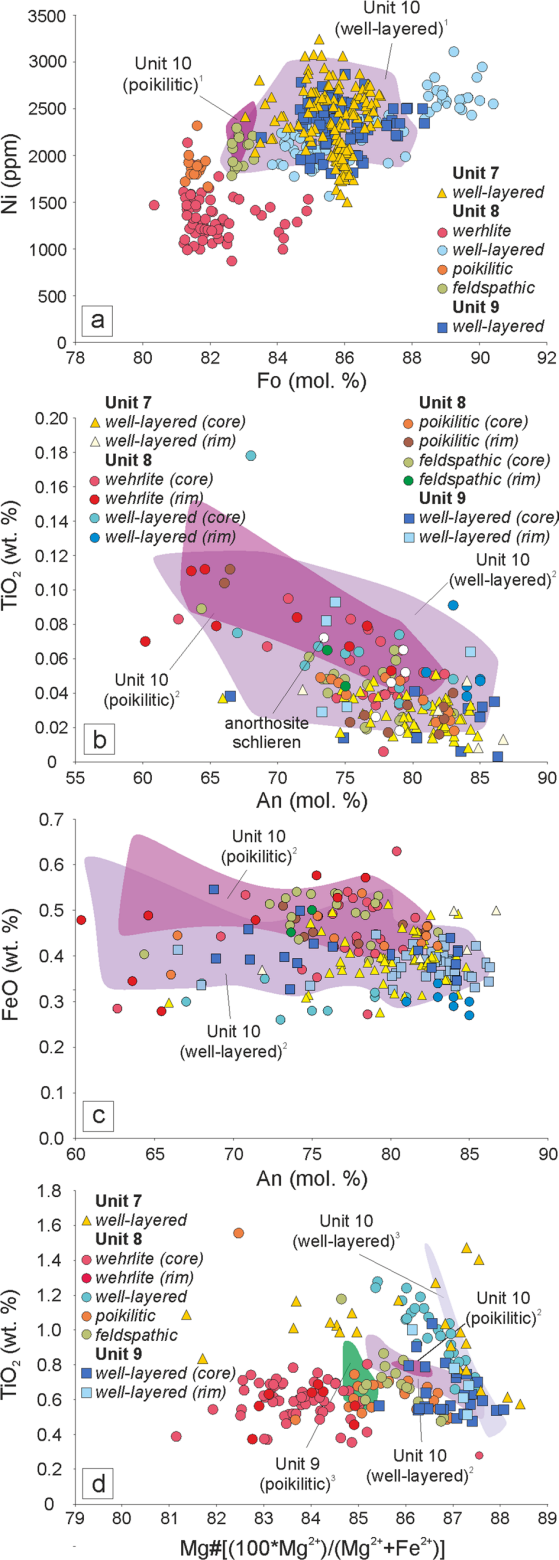
Mineral chemistry from the Unit 7, 8, and 9 peridotites. **a** Fo (mol%) versus Ni (ppm) for olivine. **b**, **c** TiO_2_ and FeO (wt%), respectively, versus An for plagioclase. **d** TiO_2_ (wt%) versus Mg# for clinopyroxene. ^1^Hepworth et al. 2017; ^2^Hepworth 2018; ^3^Holness et al. 2007

Olivine from the Unit 8 poikilitic peridotite has a very narrow, low forsterite content range (81.3–81.9 mol%), with a similarly restricted Ni content (1666–2318 ppm; Fig. 10a) and no mineral chemistry differences observed between skeletal and granular olivine morphologies. Olivine in the well-layered peridotite is highly forsteritic, with a limited range of 87–83 Fo mol%, with no mineral chemical difference between harrisitic and granular-type olivine (Fig. 10a). Nickel concentrations vary between 1501 and 3245 ppm. The composition of the Unit 8 well-layered peridotites represents the highest forsterite contents (up to 90 mol%) with correspondingly high Ni (commonly > 3000 ppm). Particularly high forsterite and Ni contents occur in olivine associated with thick Cr-spinel seams within Unit 8 (e.g. Fig. 4e). The feldspathic peridotite shows a narrow forsterite and Ni content range (83–82 mol%, 1784–2294 ppm, respectively). Olivine within the wehrlite has comparatively low forsterite content (~ 80–85 mol%) with low Ni contents (872–2137 ppm; Fig. 10a). The small rounded olivine crystals enclosed by clinopyroxene described above (e.g. Fig. 9c) have marginally lower forsterite contents than the bulk olivine (< 82.5 mol%). No chemical zoning was observed.

#### Plagioclase

Intercumulus plagioclase from the Unit 8 poikilitic peridotite displays a broad range of anorthite composition (66–83 mol%), with normally zoned crystals showing core to rim variations of 82–66 mol%. Reverse zoning is uncommon with core-to-rim variations of 74–82 mol%, respectively, while rare examples of oscillatory zoning reveal core–rim–rim values from 73 to 83 to 78 mol%, respectively. The K_2_O content is higher compared to other peridotites in Unit 8 (~ 0.16 wt%) with a high average FeO (~ 0.45 wt%; Fig. 10c). Intercumulus plagioclase from the well-layered peridotites displays a compositional range from 65 to 88 An mol% (Fig. 10b, c). Reverse zoning is common, with rim to core variations of 75–84 An mol%, respectively, while uncommon normal zonation reveals core to rim variation of 81–71 An mol%, respectively. The TiO_2_ and FeO contents do not vary between core and rim, averaging ~ 0.03 (165 ppm) wt% and 0.44 wt%, respectively. (Fig. 10b, c). Plagioclase (cumulus) in the feldspathic peridotite is compositionally similar to the intercumulus plagioclase in the poikilitic peridotite, ranging from 64 to 83 mol% (Fig. 10b, c). The abundances of K_2_O and FeO are also similar, averaging 0.16 wt% and 0.47 wt%, respectively. Normally zoned crystals have small core-to-rim variations (e.g. 79 and 75 mol%, respectively), while reverse zoning shows more pronounced variation from core to rim (e.g. 64 and 73 mol%, respectively). Plagioclase (intercumulus) in the wehrlite displays a restricted range in anorthite composition (73–79 mol%). Normal zoning is common with average core to rim variation of 78 to 69 mol%, respectively. The FeO contents are consistently high (~ 0.45 wt%), while there is a moderate increase in K_2_O from core to rim (0.07–0.14 wt%, respectively). Similarly, the TiO_2_ contents increase in the rims, with average core-to-rim variation of 0.04–0.08 wt%, respectively. The composition of cumulus plagioclase within the anorthosite schlieren in Fig. 7e is identical to intercumulus plagioclase from the wehrlite. Unzoned crystals across all peridotites typically show the highest anorthite contents and lowest TiO_2_ contents.

#### Clinopyroxene

Clinopyroxene from the Unit 8 poikilitic peridotite has an intermediary composition between other types (well-layered and feldspathic, for example), with a range in Mg# of 82–87. The Cr_2_O_3_ averages ~ 0.85 wt%, with an average TiO_2_ abundance of ~ 0.66 wt% (Fig. 10d). Clinopyroxenes analysed within the Unit 9 poikilitic peridotite by Holness et al (2007) overlap strongly with those here (Fig. 10d). Clinopyroxenes in the well-layered peridotites have wide-ranging compositions, with Mg# between 81 and 88 (Fig. 10d). The Cr_2_O_3_ content has a relatively wide range of 0.24–1.09 wt.%, with values typically 1 wt% for Units 8 and 9. The TiO_2_ content of clinopyroxene is comparatively high, consistently ~ 1 wt%, and correlating negatively with Mg# (Fig. 10d). Faint patchy zoning of TiO_2_ exists in some clinopyroxene oikocrysts without any corresponding changes in Mg# or Cr_2_O_3_. Clinopyroxene in the feldspathic peridotite has very similar compositions to the poikilitic peridotite, with marginally higher Mg# (84.6–87). This slight increase is also shown in Cr_2_O_3_ and TiO_2_, which are both comparatively increased compared to the poikilitic peridotite (~ 0.94 wt% and 0.71 wt %, respectively) (Fig. 10d). Clinopyroxene from the wehrlite has a distinctly low average Mg# (~ 83), with a subtle negative correlation with TiO_2_ (Fig. 10d). There is significant overlap in Cr_2_O_3_ between these clinopyroxenes and those from the poikilitic peridotite, though the wehrlite extends to lower values (< 0.60 wt%). Patchy zoning is apparent within clinopyroxene oikocrysts, with core-to-rim configurations difficult to discern. In optically discernible cores, the Cr_2_O_3_ is typically higher (~ 0.75 wt%) with rim values of ~ 0.40 wt%. There does not appear to be any marked variation between zones in terms of Mg# or TiO_2_, with strong overlaps between groups.

#### Cr-spinel

**Fig. 11:**
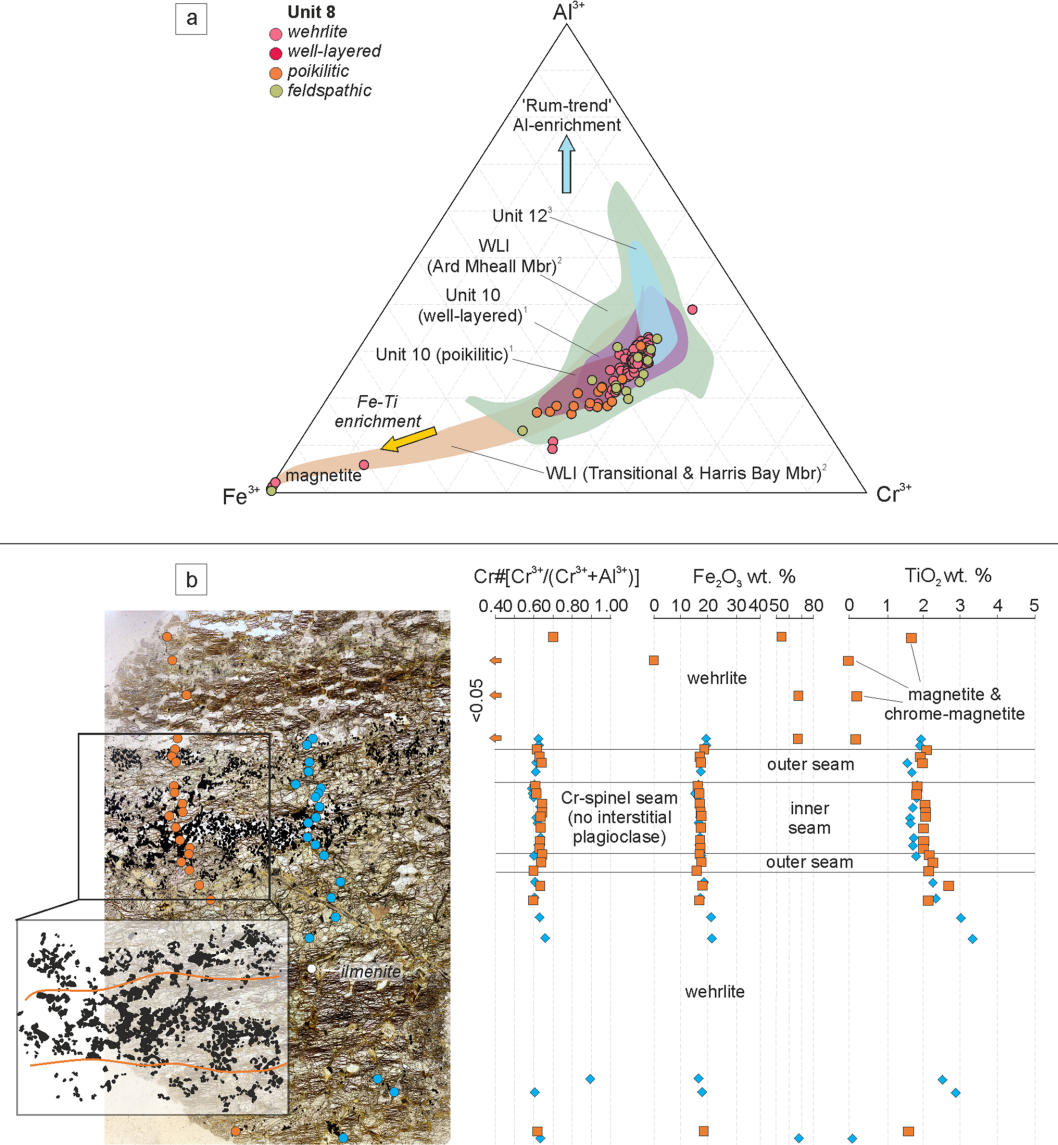
Cr-spinel mineral chemistry for peridotites in Units 7, 8, and 9. **a** Trivalent ternary diagram for Cr-spinel. ^1^Hepworth et al. 2017; ^2^Hepworth et al. 2018; ^3^O’Driscoll et al. 2010; ^4^O’Driscoll et al. 2009a. **b** Cr-spinel seam from wehrlite in Unit 8 with constant composition through the seam and into the surrounding wehrlite. Note the elevated TiO_2_ and Fe_2_O_3_ compared to seams found in the ELI (e.g. O’Driscoll et al. 2010; Hepworth et al. 2017)

There is a limited compositional range in disseminated Cr-spinel within the Unit 8 poikilitic peridotite Cr# (0.60–0.72), with a similarly limited range in Mg# (17–38). The TiO_2_ content of Cr-spinel is particularly high, averaging 4.2 wt%, with a range between 2.8 and 5.2 wt%. Ferric iron content is also high, between 14 and 31 wt%, averaging ~ 24.5 wt% (Fig. 11a). The disseminated Cr-spinel in the feldspathic peridotite displays a restricted composition of Cr# (0.58–0.73), while the Mg# of Cr-spinel is higher and more variable (10–45). The TiO_2_ content is comparatively high, but lower than that of the poikilitic peridotite (average ~ 3.1 wt%), with a similar trend for Fe_2_O_3_, averaging ~ 20 wt%, with a range of 14–34 wt% (Fig. 11a). The composition of Cr-spinel from wehrlite displays a relatively restricted composition, with a range of Cr# of 0.59–0.74 and a range of Mg# of 25–52. The TiO_2_ content is consistently high with a range between 1.60 and 5.26 wt%. The ferric iron content is also high, ranging from 12 to 28 wt%. The composition of Cr-spinel in the wehrlites is comparatively uniform from traverses across seams, for example, TiO_2_ varies by only 0.76 wt%, with Fe_2_O_3_ varying by ~ 2 wt%, with even lower variation in some seams of < 1 wt% (Fig. 11b).

## Discussion: sill emplacement and metasomatism in the Rum Eastern Layered Intrusion

### Braided geometry of peridotite sills

Harker’s (1908) original interpretation of the ELI was that it represented a series of sills (i.e. a sill complex). Many of the subsequent interpretations of the ELI were overwhelmed by principles founded upon the discovery of the Skaergaard Intrusion in East Greenland (Wager and Deer 1939). Brown’s (1956) investigation of the ELI was therefore strongly influenced by the magma chamber paradigm borne out of studying the Skaergaard Intrusion. That the RLS could have formed via sill emplacement was not explicitly revisited until the detailed re-investigation of some macro-rhythmic units in the ELI by Bédard et al. (1988). The authors were able to demonstrate from field relationships that the Unit 9 and 10 peridotites were sills, and then speculated that most (if not all) of the other ELI peridotites might also represent sills (see Renner and Palacz 1987; Bédard and Sparks 1991). Despite the conclusive evidence shown by Bédard et al. (1988) within the ELI, their sill emplacement model was underrepresented in subsequent interpretations of many aspects of the layered intrusion (Volker and Upton 1990; Holness 2005, 2007; O’Driscoll et al. 2007, b; Holness and Winpenny 2008; Latypov et al 2013; Emeleus and Troll 2014). It was not until a recent investigation of the Unit 10 peridotite that the concept of sill emplacement was used to explain the cross-cutting nature of harrisite layers, strongly suggesting that the peridotite body was constructed from numerous, small-volume sill emplacement events (Hepworth et al. 2017).

To determine whether a layer in a layered intrusion represents either a sill-like intrusion or a layer formed via crystal settling, first-order features such as lateral discontinuities or cross-cutting relationships of the overlying cumulate are typically used as criteria, as these features cannot be explained by gravity-driven or chamber floor processes (e.g. Bédard et al. 1988; Tegner and Robins 1996; Hepworth et al. 2017; Latypov et al. 2017). Within Units 7, 8, and 9, the criteria of sill emplacement are fulfilled amply, with both peridotite subtypes exhibiting cross-cutting relationships with the overlying allivalite. The Unit 7 well-layered peridotite, for instance, displays upward-oriented apophyses at varied scales (from centimetre to metre scale) that cross-cut the overlying troctolite (Fig. 5b–d). The apophyses bear some resemblance to the so-called ‘finger structures’ described by Butcher et al. (1985), although the authors dismissed injection of peridotite into the troctolite as they deemed the fingers too small for injection and instead argued for replacement by metasomatism. The Unit 9 well-layered peridotite displays larger dome-like structures of harrisite that cross-cut the troctolite and perhaps into the Unit 10 peridotite (Fig. 2). Unit 8, a more complex peridotite body, exhibits broad cross-crossing features of the poikilitic peridotite, where it is higher or at the same stratigraphic level as the supposedly overlying allivalite (Fig. 4a). Lateral discontinuity of the peridotite bodies is also common across all three units (particularly those found in the north of the study area). The poikilitic peridotites thin northward until they become absent from the succession (e.g. Fig. 2). Hepworth et al. (2017) did not report finding the Unit 10 poikilitic peridotite (their ‘’upper peridotite’’) around Barkeval, suggesting it had already vanished from the succession, as observed in this study with Units 7, 8, and 9. The well-layered peridotites display the opposite relationship and are thickest around Barkeval and thinnest around Hallival, roughly coincident with distance from the LLF feeder zone (Fig. 1b, c). The Unit 8 well-layered peridotite is actually missing between Units 7 and 9 around Barkeval as shown on Fig. 2 but is present several hundred metres south where the boundary chromitite occurs (e.g. O’Driscoll et al 2010). Furthermore, there are numerous locations where large-scale peridotite coalescence is observed between well-layered peridotites (something only implicit in previous studies; e.g. Young 1984; Butcher et al 1985; Bédard et al 1988). For example, the well-layered peridotites of Units 9 and 10 coalesce around Barkeval, with no obvious textural or mineralogical variation between the two peridotites at the supposed boundary (Fig. 6a). Similar observations are also present between Units 8 and 9 (Fig. 2).

**Fig. 12:**
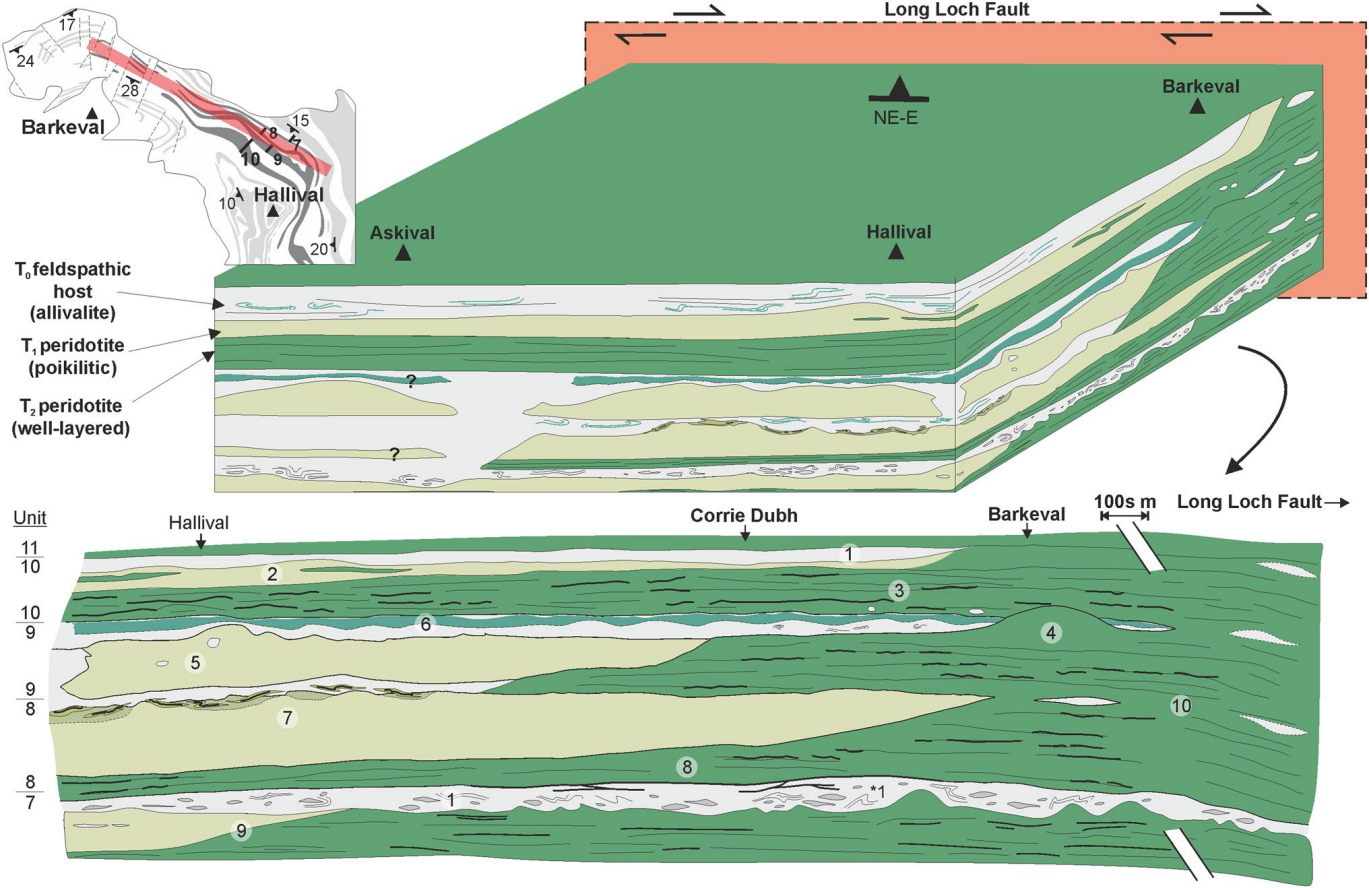
Three-dimensional block diagram representing the field relationships of Units 7–11 from observations in this study and by others (Bédard et al. 1988; Hepworth et al. 2017; Hepworth 2018). ‘*T*_0–2_’ refers to the relative timing of the three components during the formation of the ELI. 1 *T*_0_ pre-existing feldspathic cumulate (i.e. troctolite and gabbro) including the 1* deformed and anorthosite schlieren-rich Unit 7 troctolite marker horizon. 2 T_1_ poikilitic peridotite, typically absent with proximity from the LLF. 3 T_2_ well-layered peridotite with abundant Cr-spinel seams, occurring abundantly with proximity to the LLF, built up of small volume replenishment events of harrisite and granular-textured peridotite. 4 Cross-cutting relationships in well-layered peridotite, i.e. harrisite dome in Unit 9 (Fig. 2). 5 Cross-cutting relationships in the poikilitic peridotite, including incorporated troctolite blocks, i.e. Unit 9 at Hallival. 6 The clinopyroxene-rich wavy horizon in Unit 9, an example of metasomatism from the intrusion of peridotite. 7 Metasomatism of the poikilitic peridotite and formation of the feldspathic peridotite and wehrlites in Unit 8 as part of the T_2_ replenishment episode. 8 The discontinuous chromitite seam between Unit 7 and 8. 9 Laterally thinning terminus of well-layered peridotite sills away from the LLF, toward Hallival. 10 Braided, interconnected well-layered peridotites closer to the LLF where poikilitic peridotite is typically absent, alongside highly discontinuous troctolites

Our observations for the peridotites within Units 7, 8, and 9 cannot be explained by gravity-driven or chamber floor processes traditionally applied to layered intrusion formation and have more in common with dyke and sill complexes. The features are therefore interpreted as first-order evidence for sill emplacement. This is in line with previous observations of Unit 9, where this study serves to highlight the lateral complexity of the layers examined by Bédard et al. (1988) and further support their hypothesis. The explicit recognition of peridotite coalescence between multiple units serves as strong evidence for sill emplacement, suggesting that the peridotites are connected in three dimensions by bridges of cumulate (i.e. braiding or anastomosis). Our observations are combined with previous field evidence and illustrated in Fig. 12 for Units 7–10. The similarity between texture and mineralogy of the peridotites within coalesced peridotites and both underlying and overlying layers points towards a single event, i.e. the Unit 9 and 10 peridotites represent the same peridotite body separated by pre-existing allivalite (Fig. 12). This conclusion could be extended to all well-layered peridotites in the ELI which share very close textural, mineralogical, and mineral chemical characteristics and may represent similar parental magmas (e.g. Fig. 10a, d).

### Incremental emplacement of peridotite sills

The construction of Units 7, 8, and 9 peridotites via sill emplacement is important for understanding the broad-scale construction of the ELI; however, like many intrusive bodies (e.g. granitoids), there is an inherent issue with accommodation space when emplacing up to 60 m of magma (e.g. the Unit 10 peridotite) into any pre-existing lithology (Annen et al. 2015). The peridotite portions of Units 7, 8, and 9 each contain at least two peridotites, much like Unit 10 described by Palacz and Tait (1985) and Hepworth et al. (2017). The spatial relationship between the two peridotites is complex laterally. For example, the poikilitic peridotites are typically absent closer in the north (i.e. around Barkeval), where the well-layered peridotite dominates (e.g. Fig. 2). Conversely, the well-layered peridotite is thinner to the south (i.e. around Hallival), where the poikilitic peridotite can be > 20 m thick (Fig. 12). This is important, as the poikilitic peridotite of Unit 10 was reported to pre-date the well-layered peridotite based upon metasomatism at the contact of the two peridotites (Hepworth et al 2017). If the well-layered peridotite post-dates the poikilitic peridotite, we might expect a greater abundance of the peridotite relative to the host lithology nearer to the source (e.g. Figs. 1c and 2). It might also be expected that the well-layered would thin out away from the source, something noted for the well-layered peridotites in all three units (Fig. 12; see also Bédard and Sparks 1991). In this scenario, well-layered peridotites effectively underplate the poikilitic peridotite as it propagates away from the source (Huppert and Sparks 1980). An explanation whereby a buoyant poikilitic peridotite forms latest is not corroborated by our observations, as the poikilitic peridotite tapers out towards the source. If the poikilitic peridotite was younger than the well-layered peridotite, we would not expect it to be missing from source proximal locations like Barkeval (Fig. 12). Based on this evidence, we suggest that the two peridotite subtypes within Units 7, 8, and 9 represent at least two temporally separate replenishment events, with the well-layered peridotite intruding after the intrusion of the poikilitic peridotite (Hepworth et al 2017). This conclusion is supported by the fact that both subtypes are geochemically distinctive (Fig. 10a, d). The mineral chemistry of olivine, plagioclase, and clinopyroxene suggests that the poikilitic peridotite is more evolved than the well-layered peridotite (e.g. Palacz and Tait 1985; Hepworth et al 2017).

Further to the two distinct peridotite bodies discussed above, the well-layered peridotites consist of numerous harrisite layers. Hepworth et al. (2017) showed that harrisite layers found in the Unit 10 peridotite represent sills based largely on their topology. For example, they highlighted the presence of small-scale features such as upward-oriented apophyses, bifurcation, and lateral discontinuities, which must have formed within the crystal mush and not upon the magma chamber floor (see also Donaldson 1982; Hepworth et al. 2018). The harrisite layers found throughout the well-layered peridotites in this study display the same features as those described in the Unit 10 peridotite (Fig. 4d; 5f and 6e). The well-layered peridotites of Units 7, 8, and 9, must, therefore, have been constructed from numerous, small-volume picrite sills emplaced directly into the crystal mush. The composite textures of many harrisite layers in Unit 7 shown in Fig. 5f, for example, further support this hypothesis, with the coarsest-grained crystals along the margins of the harrisite sills where the temperature difference will be greatest, promoting deeper degrees of undercooling and crystal growth than during emplacement of the centre (Donaldson 1976). Many (if not all) of the granular-textured peridotites represent finer grained sills unable to form large skeletal crystals (e.g. Hayes et al. 2015a; b; Hepworth et al. 2017, 2018). The abundance of Cr-spinel seams found within the well-layered peridotite further attests to this process and points to a greater number of emplacement events than that highlighted by the number of harrisite sills (Figs. 3a, b, 4e, 5b and 6d).

The formation of layered intrusions via sill emplacement is likely more widespread than suggested in the literature and not limited to thin peridotite layers (e.g. Karykowski and Maier 2017). The protracted emplacement of small-volume sills into a developing crystal mush will inevitably have complicated the cooling history of the complex as a whole. Many crystal mushes could be kept hotter for longer periods of time if the rate of intrusion is high, constantly buffering the temperature of a system that should be cooling down at a constant rate (Cashman et al. 2017). It is also possible that if the intrusion rate is low, layered intrusions are allowed to cool significantly before being rejuvenated by replenishment, potentially disrupting the recorded thermal history and forming the types of metasomatic horizons we observe within the ELI (e.g. Holness et al. 2007; Leuthold et al. 2014). Our findings serve to demonstrate that features such as upward-oriented apophyses and laterally thinning or thickening layers (or packages of cumulate) can be used to infer sill emplacement (and incremental emplacement) within layered intrusions irrespective of the scale at which they occur. Many of the smaller-scale features present in the ELI can be compared directly to much larger field observations in much larger layered intrusions, formed from much larger volumes of magma input (e.g. Bushveld or Stillwater Complexes; Latypov et al 2013; Mungall et al. 2016; Wall et al. 2018; Latypov 2019; Scoates et al 2019).

### Formation of the macro-rhythmic units

The macro-rhythmic units of the ELI were established by Brown (1956), who suggested the cyclicity formed via open-system magmatism, with each unit representing a single replenishment event fractioning through olivine–plagioclase ± clinopyroxene. The evidence presented here is not compatible with this concept. Notably, the identification of cross-cutting and coalescence between units question the relevance of the macro-rhythmic (or cyclic) units. The past interpretations of cyclic successions of peridotite, troctolite, and gabbro are fundamentally rooted in the basaltic fractionation sequence (olivine–plagioclase–clinopyroxene). In our opinion, this preconception has resulted in a bias toward assuming that each unit evolved separately. There is no reason why any of the lithologies in the ELI need to be related by magma fractionation in the traditional sense (i.e. crystal settling), and, given the evidence now presented here and by others (e.g. Renner and Palacz 1987; Bédard et al. 1988; Hepworth et al. 2017), we argue that the relationship between peridotite and allivalite on Rum is simply an artefact of the juxtaposition of sills and the host rock (i.e. gabbroic rocks). As such, the unit divisions should not be numbered in strict stratigraphic groups (i.e. as units) which the field observations presented here have shown to become unclear laterally. If they are to be given arbitrary numbers, it should be separated based on lithology, e.g. peridotite 1, troctolite 1, peridotite 2, etc. The current grouping into units implies a genetic relationship between the individual members, which can skew interpretation. An example of where this has potentially skewed petrogenetic interpretation is the misinterpretation of peridotite coalescence and allivalite truncation as faulting within the north of the study area (Emeleus 1994; Fig. 1c). Future work should approach the interpretation of cyclic sequences of cumulate in layered intrusions with caution (cf. Latypov et al. 2017). The varying members within so-called cyclic sequences could very well be intercalated sills and the pre-existing cumulate, irrespective of scale (e.g. Renner and Palacz 1987; Karykowski and Maier 2017). More complex sequences of cumulate might represent the pre-existing cumulate, the intruding sill, and an intermediary (hybrid) lithology formed from the reaction between the two components, which would appear to link the layers by magma fractionation (e.g. Wadsworth 1961; Tegner and Robins 1996; Hepworth et al. 2018).

## Laterally oriented metasomatism in peridotite cumulates

The Unit 8 peridotite in the south of the study (around Hallival) is made up of four distinct peridotite subtypes (Fig. 3a). We have previously discussed the relationship between two of the subtypes found within all three units: the well-layered and poikilitic peridotites. Here, we posit a mechanism for the formation of the two unusual subtypes found at the top of the Unit 8 peridotite: the feldspathic peridotite and wehrlite (Fig. 4b, c, 7a).

### The feldspathic peridotite as a metasomatic horizon

The feldspathic peridotite is a discontinuous body of clinopyroxene-poor peridotite occurring between the poikilitic peridotite and troctolite at the very top of the peridotite sequence (Fig. 4b, c). The boundary between the feldspathic peridotite and overlying troctolite is sharp and undulose only over a large scale (several metres). The boundary between the feldspathic peridotite and underlying poikilitic peridotite is complex and locally undulose (Fig. 4b). There are no macroscopic differences in the appearance of the two peridotites besides the reduced concentration of clinopyroxene in the feldspathic peridotite. The boundary between the two lithologies depicted in Fig. 4b is very similar to the geometry of the clinopyroxene-rich ‘wavy horizon’ found within the Unit 9 allivalite (Holness et al. 2007). It is possible, based on these key features, that the feldspathic peridotite represents a zone of metasomatism. If this was the case, the most likely reactive liquids entering the crystal mush in the ELI are picritic (Greenwood et al. 1990; Upton et al. 2002; Leuthold et al. 2015). The olivine-normative picrites would have been capable of preferential dissolution of the crystal mush, i.e. dissolving clinopyroxene and plagioclase without olivine (Donaldson 1985). There are several observations that highlight the interaction with primitive (or picritic) magma. For example, the feldspathic peridotite consists of cumulus-textured plagioclase laths not observed in any of the other peridotites (Fig. 8f). The presence of normal and reverse zoning within many of these crystals strongly suggests recrystallisation by reactive liquid flow (*cf.* O’Driscoll et al. 2009a; Leuthold et al. 2014; Hepworth et al. 2017). The olivine found within the feldspathic peridotite is more forsteritic than adjacent lithologies, pointing to the interaction with olivine-normative magma (Fig. 10a). The composition of olivine in the feldspathic peridotite also overlaps with olivine found within a metasomatic horizon described by Hepworth et al. (2017) in the Unit 10 peridotite (Fig. 10a). Furthermore, the presence of more Mg(+ Al)-rich spinel within the feldspathic peridotite attests to the interaction with more primitive magma such as picrite (Fig. 11a) (Bell and Claydon 1992; Lenaz et al. 2011; Leuthold et al. 2015).

The emplacement of large sills has been put forward to explain zones of infiltration metasomatism found within crystal mushes in the RLS (e.g. Holness et al. 2007; Leuthold et al. 2014; Hepworth et al. 2017; 2018). In these examples, the sill, typically picritic, is always in contact with the metasomatised crystal mush. However, the only peridotite sill bounding the feldspathic peridotite is the poikilitic peridotite, which cannot be have formed the reaction zone because the reaction zone (i.e. the feldspathic peridotite) occurs *within* the poikilitic peridotite (Fig. 4b, c). This scenario is only plausible if we assume the protolith of the feldspathic peridotite has been completely digested elsewhere, and, given the similarity between the two peridotites and resemblance to other reaction zones, this is considered unlikely (e.g. Fig. 4b, 6a). The only alternative is that the wehrlites caused the metasomatism.

The wehrlites are sill-like bodies that cross-cut the feldspathic peridotite and overlying troctolite, post-dating both lithologies, akin to the well-layered peridotites. The wehrlites also host Cr-spinel seams (Fig. 7b, c). But why are these peridotites enriched in clinopyroxene if they are temporally associated with the clinopyroxene-poor, well-layered peridotite (i.e., picrites)? This is particularly important where Cr-spinel is found (e.g. Fig. 7c). Spinel should not co-crystallise with clinopyroxene as they do not normally share a cotectic (*cf*. Onuma and Tohara 1983). In fact, excess silica in the melt (when crystallising clinopyroxene) may trigger reabsorption of spinel (Morse 1980). The composition of Cr-spinel found within seams in the wehrlite is enriched in Fe^3+^ and TiO_2_ (Fig. 11a, b), with crystals of ferroan chromite and Cr-magnetite observed (Fig. 11b), strongly associated with metasomatised examples (Bell and Claydon 1992; O’Driscoll et al. 2009a; Leuthold et al. 2015). The composition of spinel in seams found within the RLS varies above and below the seam, reflecting varying conditions of crystallisation (O’Driscoll et al 2009a; 2010; Hepworth et al 2017). A traverse through one of the wehrlite-hosted Cr-spinel seams reveals constant spinel compositions (Fig. 11b). This would suggest that either the conditions of crystallisation were uniform in the footwall, hanging wall, and seam, or that the whole rock had been modified uniformly. As spinel and clinopyroxene do not often co-crystallise, we must assume the rock has been modified uniformly (and severely). Our conclusion is supported by the anhedral (e.g. amoeboidal) morphology of many Cr-spinel crystals within the seams (Fig. 9a, b), common in modified Cr-spinel seams (Vukmanovic et al 2013). In other words, the spinel predates (and was modified by) clinopyroxene growth. There is also evidence for severe modification of the olivine found within the wehrlites, further suggesting disequilibrium between co-existing phases. The clinopyroxene in the wehrlite is commonly zoned, with a patchy and oscillatory configuration suggestive of multiple phases of reactive liquid flow through the crystal mush (Fig. 9c). The oikocryst in Fig. 9c also displays a texture whereby zoned oikocrysts of clinopyroxene contain scarce, small, and rounded olivine inclusions. Barnes et al. (2016) argued that similar textures, found in layered pyroxenites from the Ntaka Ultramafic Complex in Tanzania, were formed via secondary reaction, removing pre-existing mineralogy (e.g. plagioclase and olivine) and precipitating pyroxene, with similar textures observed in peridotites metasomatised by clinopyroxene-saturated melts in the Rum WLI (Hepworth et al 2018).

Our observations suggest that clinopyroxene was not in equilibrium with the other constituent phases in the wehrlites (olivine and spinel). If clinopyroxene is subtracted from the mineralogy, we are left with features typically associated with the well-layered peridotites (including Cr-spinel seams). As such, we argue that the wehrlites represent heavily modified picritic sills emplaced along the boundary of the allivalite and peridotite (e.g. Fig. 4c). As the only candidate for picritic magma necessary for infiltration metasomatism, we suggest that these sills were responsible for the metasomatism of the feldspathic peridotite protolith, prior to being modified by a reactive, clinopyroxene-oversaturated melt and transformed into wehrlites (see below) (Fig. 14).

### The origin of clinopyroxene-oversaturated melt and formation of the feldspathic peridotite metasomatic horizon

**Fig. 13:**
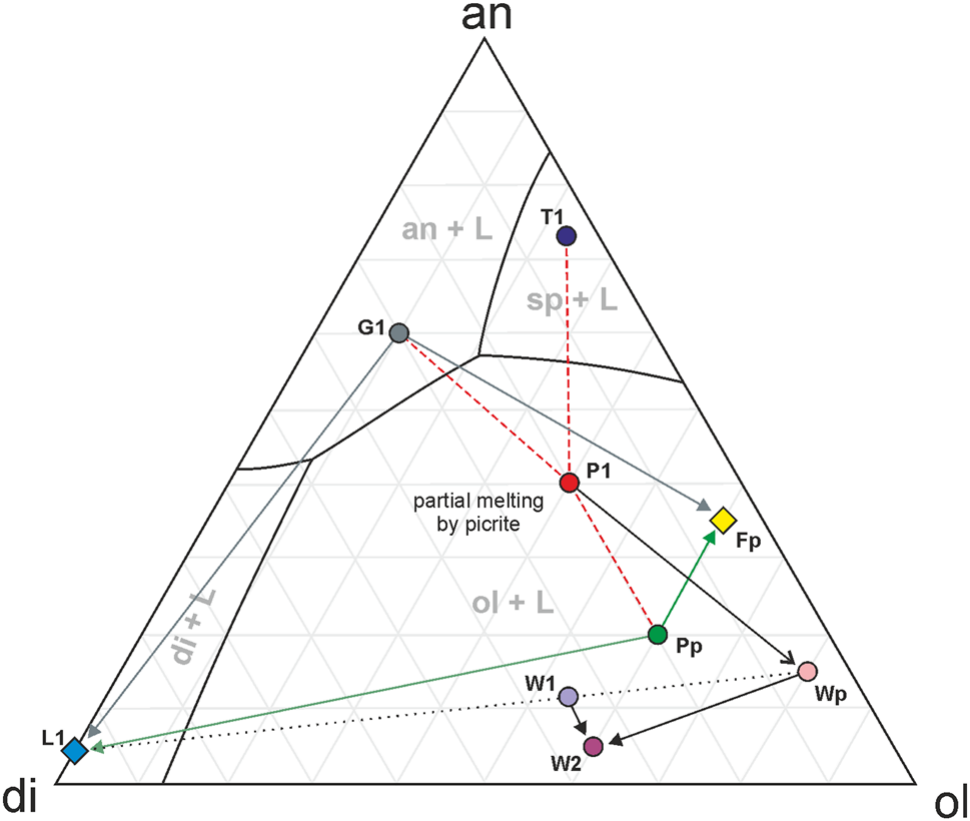
Schematic, pseudo-ternary diagram for the reactions posited in the discussion on metasomatism within the Unit 8 peridotite. T1 (troctolite), G1 (gabbro), P1 (picrite), L1 (liberated clinopyroxene-oversaturated melt), Pp (poikilitic peridotite), Fp (feldspathic peridotite), Wp (well-layered peridotite), W1 (binary mixed wehrlite), W2 (observed wehrlite). See text for full description

The source of the clinopyroxene-oversaturated melt that modified the picrite sills must have originated from the protolith of the feldspathic peridotite. If this was not the case, the percolation of clinopyroxene-oversaturated melt through the feldspathic peridotite during the modification of the wehrlites would have caused similar modification to that in the wehrlites and overprinted the markers for picrite interaction discussed above. There are three potential lithologies for the protolith of the feldspathic peridotite and source of the clinopyroxene-oversaturated melt that formed the wehrlites: the overlying troctolite, a gabbro, or the underlying poikilitic peridotite. Leuthold et al (2014) performed a series of partial melting and mixing calculations to explain the origin of several different cumulates with clinopyroxene oikocrysts from the Unit 9 allivalite, focused on the role of reactive liquid flow (*cf* Leuthold et al. 2015*.*). We can couple these helpful parameters with our own observations to make some inferences about the types of reactions and degrees of partial melting responsible for our metasomatised cumulates. The reactions can be illustrated using schematic pseudo-ternary diagrams (Fig. 13). The intruding magma (P1) would have had an equivalent composition to an aphyric parental picrite (Upton et al. 2002; Holness et al. 2007). A troctolite protolith (T1) would have had a composition similar to those used by O’Driscoll et al. (2009a). Leuthold et al. (2014) argued that some olivine-rich cumulate in the Unit 9 allivalite could have formed by high degrees of partial melting (> 70%) and dissolution of troctolites and gabbros. However, the troctolite does not contain enough clinopyroxene to account for the clinopyroxene-oversaturated melt which must have come from the protolith, even if the reaction with an intruding picrite sill managed to concentrate olivine to account for what is presented in the feldspathic peridotite (60–70 vol%). A reaction of picrite magma and gabbro, however, has been used within the ELI to explain high concentrations of clinopyroxene in nearby cumulate (e.g. Holness et al. 2007; Leuthold et al. 2014). The gabbro (G1) would have enough clinopyroxene to account for the reactive, clinopyroxene-oversaturated melt present in the wehrlites (L1) and would leave behind troctolite after dissolution (Fig. 4c). The cumulus nature of the plagioclase, would, on first glance, support this view (Fig. 8f). But for the gabbro to accumulate the 60–70 vol% olivine within the ~ 1 m-thick feldspathic peridotite (Fp), we would expect large degrees of partial melting (> 70%; Leuthold et al. 2014) over an area of tens of metres (Fig. 4b). The metasomatism of that much cumulate would also produce a plagioclase-rich melt equivalent to the difference in plagioclase between the feldspathic peridotite and gabbro over ~ 1 m of cumulate (~ 25%) (Fig. 13). The only evidence for plagioclase-rich melt is scant veins (Fig. 7a). Furthermore, the metasomatism of troctolite or gabbro cannot account for the occurrence of harrisite (skeletal) olivine within the feldspathic peridotite (Fig. 8f). The only adjacent alternative is the poikilitic peridotite. Metasomatism of the poikilitic peridotite by the olivine normative P1 magma would preferentially dissolve clinopyroxene and plagioclase (e.g. Donaldson 1985; Leuthold et al. 2014). This reaction would produce the L1 clinopyroxene-oversaturated melt by dissolving out most of the clinopyroxene from the poikilitic protolith and form the feldspathic peridotite restite (Fp). The close association of clinopyroxene chemistry between the feldspathic peridotite and poikilitic peridotite supports this argument (Fig. 10d). The similar plagioclase abundances between both peridotites suggests plagioclase-rich melt was not mobilised into the crystal mush like clinopyroxene (Fig. 13). Any plagioclase-rich melt that was mobilised might explain the presence of normal zoning and intercumulus plagioclase within the feldspathic peridotite (Fig. 8f). Lastly, if the P1 magma is temporally related to the well-layered peridotites, the emplaced sills would, if not metasomatised, crystallise to form well-layered peridotite equivalents (Wp). This process had evidently started prior to metasomatism, given the occurrence of Cr-spinel seams within the wehrlites (Fig. 7b, c). The wehrlites can, therefore, be considered a mixture between L1 and Wp (i.e. metasomatised well-layered peridotite). A binary mixing line constructed between L1 and Wp to form a 50–60% hybrid composition between the two components (W1), very close to the observed W2 wehrlite composition, strongly supports our hypothesis.

**Fig. 14:**
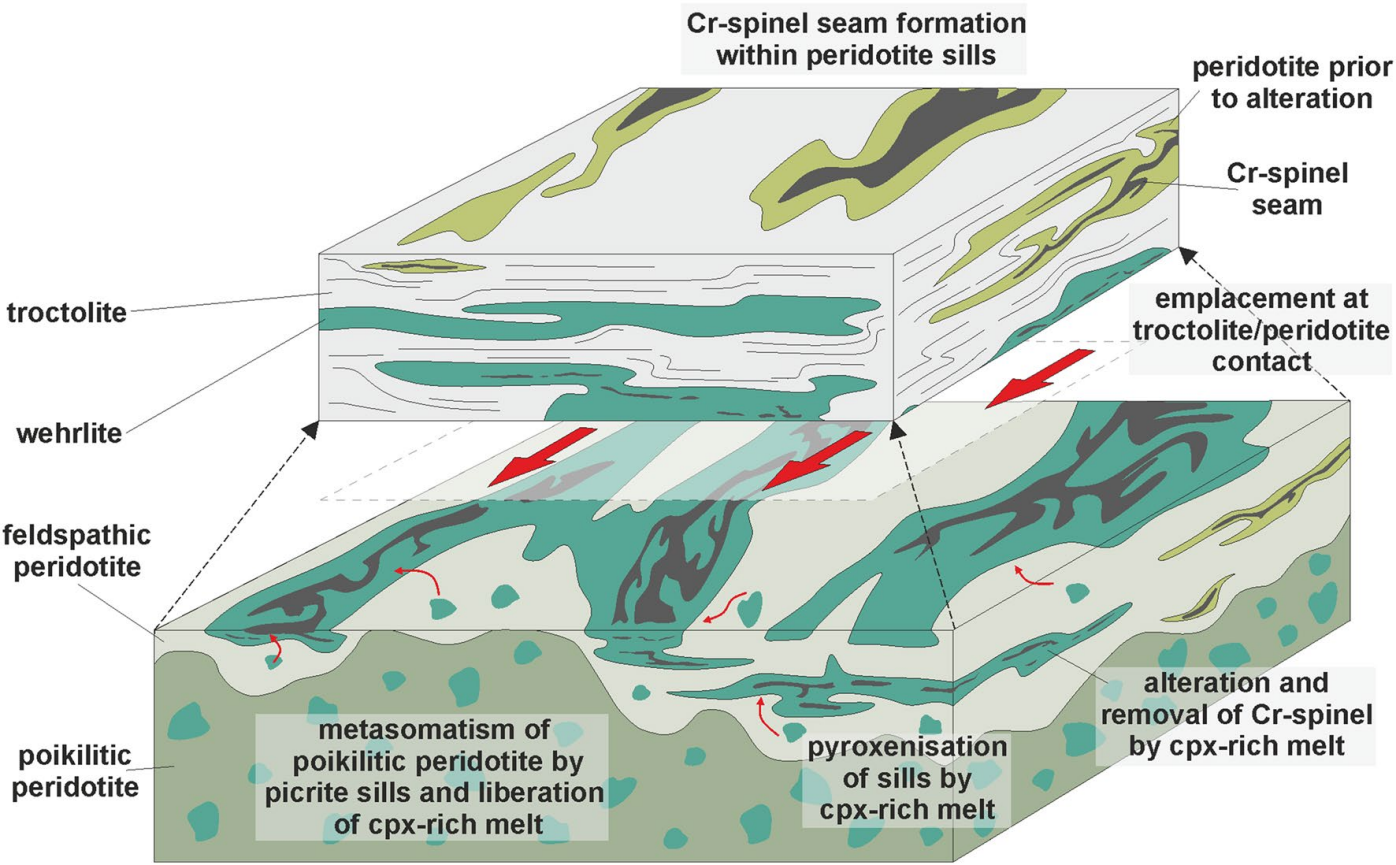
Expanded block diagram illustrating the formation of the feldspathic peridotite and wehrlites in the Unit 8 peridotite by infiltration metasomatism. See text for discussion

In summary, we argue that the protolith of the feldspathic peridotite and source of the clinopyroxene that formed the wehrlites was the poikilitic peridotite. A conceptual model for this process is presented in Fig. 14 and summarised here. Thin picrite sills were emplaced into the crystal mush at the boundary between the peridotite and allivalite, roughly coeval to the well-layered peridotites. The hot, primitive picritic magma metasomatised the surrounding poikilitic peridotites and liberated a clinopyroxene-oversaturated melt, forming the feldspathic peridotite restite (e.g. Leuthold et al. 2014). The intruded picrite sills would have acted as zones of weakness and lower differential stress, creating ideal zones for the collection of melt, heavily modifying the original mineralogy and forming the wehrlites present now (Figs. 4b, c, 7a). The zoning identified within some of the clinopyroxene oikocrysts suggests that reactive liquid flow played an important role in the formation of the wehrlites (Leuthold et al. 2014). We cannot discount completely the metasomatism of some of the troctolite, as wehrlite sills penetrate the troctolite above the boundary with the peridotite in some places. A clinopyroxene ‘wavy horizon’ was reported by Sides (2008) in the Unit 8 allivalite, suggesting that metasomatism of the allivalite by the emplacement of the poikilitic peridotite had already removed clinopyroxene and reprecipitated it higher in the succession. Any subsequent reactive event would therefore have had only minimal effect on the troctolite in terms of clinopyroxene redistribution (*cf*. Holness 2005).

The type of metasomatism that formed the feldspathic peridotite and wehrlites in the Unit 8 peridotite occurs laterally through the crystal mush rather than by vertically oriented models traditionally applied to layered intrusions (e.g. Holness et al 2007; Leuthold et al 2014; Hepworth et al 2017; O’Driscoll and VanTongeren 2017). There was no evidence found in this study for vertical migration of melt, such as ponding or the formation of poikilitic gabbros or clinopyroxene pipes (Leuthold et al. 2014; Hepworth et al 2017). It stands to reason that since the emplacement of sills occurs along the boundaries of contrasting lithologies, infiltration metasomatism will follow the same planes of weakness (i.e. parallel to layering). Another example of this process might be the unusually clinopyroxene-rich horizon at the top of the Unit 9 well-layered peridotite shown in Fig. 6c. The clinopyroxene zone bifurcates along the strike, suggesting that lateral migration played a key role in the migration of this clinopyroxene-saturated melt, and not just vertical ascent (or descent) through the crystal mush (e.g. Holness 2005, 2007; Holness et al 2007).

The process of laterally oriented metasomatism in cumulates is an important avenue for future research, particularly when elucidating the construction of cumulate sequences, many layers of which might have evidence for metasomatism without an obvious association with sills (Hepworth et al. 2018). The process is likely more important if the sequences display evidence for mechanical deformation, allowing for the creation of exploitable pathways for sills and reactive melts (Volker and Upton 1990; Tegner and Robins 1996; Humphreys and Holness 2010; Namur et al. 2013; Hepworth et al. 2017, 2018). Although this process is apparently most obvious in the presence of clinopyroxene-rich cumulates, any cumulate layer within a sequence that displays a sudden elevation (or reduction) in any of the typical minerals should be investigated for metasomatism, irrespective of an apparent association with nearby sills. Anorthosite layers that occur sporadically throughout many cumulate sequences are an ideal candidate for this type of process, especially if the layer is not already known to be associated with a sill (e.g. Latypov et al. 2015, 2017; Boudreau 2016; Mungall et al. 2016; Wall et al. 2018).

## Conclusion

The conceptualisation of magma chambers as large bodies of crystal-poor magma is perhaps most deeply rooted in the study of layered intrusions. Evidence has been presented in this study that has been used to argue against the classic paradigm, suggesting instead that the ELI represents a series of sills emplaced into pre-existing feldspathic cumulate (gabbro or troctolite). The recognition of coalescence between supposedly disparate peridotite bodies and cross-cutting relationships with overlying cumulate attests incontrovertibly to this model, ultimately questioning the validity of the magma chamber paradigm in the ELI. Furthermore, our study emphasises the importance of incremental construction as a mechanism for the development of the peridotites. The occurrence of harrisite layers (and Cr-spinel seams) throughout the peridotites of the ELI strongly suggests much of the complex was built of numerous, small-volume replenishment events emplaced directly into the crystal mush. Our study stresses the relationship between sill emplacement and metasomatism (or reactive melt flow) within the crystal mush and makes special note of the orientation of reactive flow, which occurs primarily along the same planes of weakness exploited by the invading sills (i.e. laterally). Lastly, the formation of layering and the configuration of so-called cyclic units within layered intrusions might be better explained by the concepts put forward in this study. Here, stratigraphy is not an indicator of relative age, nor does it imply genetic relationships between adjacent cumulates. A sequence of layers, irrespective of repetition, could represent an array of sills, the pre-existing lithology, and hybrid lithologies formed from the interaction between the two components. The ELI is unlikely to be unique with respect to its construction. Many (if not all) layered ultramafic sequences found in the structurally low parts of layered intrusions could have formed from the incremental emplacement of sills and metasomatism of surrounding cumulate (e.g. Stillwater Complex, USA). The precious metals found within peridotite-hosted chromitite seams must, therefore, have formed within the crystal mush, with important implications for currently exploited deposits of peridotite-hosted ore.

## Electronic supplementary material

Below is the link to the electronic supplementary material.

Supplementary file1 (XLSX 377 kb)
